# The trend and spatial spread of multisectoral climate extremes in CMIP6 models

**DOI:** 10.1038/s41598-022-25265-4

**Published:** 2022-12-05

**Authors:** Oluwafemi E. Adeyeri, Wen Zhou, Xuan Wang, Ruhua Zhang, Patrick Laux, Kazeem A. Ishola, Muhammad Usman

**Affiliations:** 1 School of Energy and Environment, City University of Hong Kong, Kowloon, Hong Kong, SAR China; 2 Department of Atmospheric and Oceanic Sciences & Institute of Atmospheric Sciences, Fudan University, Shanghai, China; 3 Center for Ocean Research in Hong Kong and Macau (CORE), Hong Kong, China; 4 Institute for Meteorology and Climate Research Atmospheric Environmental Research, Karlsruhe Institute of Technology, Campus Alpine, Germany; 5 Irish Climate Analysis and Research UnitS (ICARUS), Department of Geography, Maynooth University, Maynooth, Ireland; 6 School of Engineering, Faculty of Science Engineering and Built Environment, Deakin University, Geelong, Australia

**Keywords:** Climate sciences, Environmental social sciences

## Abstract

Climate change could exacerbate extreme climate events. This study investigated the global and continental representations of fourteen multisectoral climate indices during the historical (1979–2014), near future (2025–2060) and far future (2065–2100) periods under two emission scenarios, in eleven Coupled Model Intercomparison Project (CMIP) General Circulation Models (GCM). We ranked the GCMs based on five metrics centred on their temporal and spatial performances. Most models followed the reference pattern during the historical period. MPI-ESM ranked best in replicating the daily precipitation intensity (DPI) in Africa, while CANESM5 GCM ranked first in heatwave index (HI), maximum consecutive dry days (MCCD). Across the different continents, MPI-LR GCM performed best in replicating the DPI, except in Africa. The model ranks could provide valuable information when selecting appropriate GCM ensembles when focusing on climate extremes. A global evaluation of the multi-index causal effects for the various indices shows that the dry spell total length (DSTL) was the most crucial index modulating the MCCD for all continents. Also, most indices exhibited a positive climate change signal from the historical to the future. Therefore, it is crucial to design appropriate strategies to strengthen resilience to extreme climatic events while mitigating greenhouse gas emissions.

## Introduction

Global climate models (GCMs) are critical for understanding climate change. Still, their coarseness restricts their usefulness for creating climate change adaptation and mitigation policies, especially at regional scales where climate change impacts are more pronounced^1^. Over the past two decades, many world regions have experienced increased intensity and frequency of extreme climate events. These extreme events have been attributed to climate change^2–5^. Nevertheless, past generations of GCMs predicted a rise in such severe occurrences^6–8^.

Coupled Model Intercomparison Project Phase 6 (CMIP6)^9^ provides significant advantages that combine representative concentration pathways (RCPs) with shared socioeconomic pathways (SSPs), model advancement, and better modelling of synoptic processes^8,10–14^. However, as these GCMs become more widely available, it is critical to continue evaluating their ability to represent extreme climatic events worldwide.

Seneviratne et al.^7^ reported more heavy rainfall episodes, decreased cold extremes, and increased daily temperature extremes globally. Adeyeri et al.^2^ observed that warm spell duration and the frequency of warm days and nights exhibited statistically significant positive trends over the transboundary Lake Chad Basin. Ge et al.^13^ investigated future changes in precipitation extremes over Southeast Asia. Chen et al.^15^ evaluated and compared CMIP6 and Coupled Model Intercomparison Project Phase 5 (CMIP5) model performance in simulating extreme seasonal precipitation in the western North Pacific and East Asia. Ridder et al.^16^ reported future changes in return periods for the co-occurrence of heatwaves, drought, extreme winds, and precipitation based on a CMIP6 multimodel ensemble.

Dike et al.^17^ evaluated seasonal precipitation extremes using CMIP6 and reported an increase in the severity of projected dry spells over Central Asia. Collazo et al.^18^ considered CMIP6 models' ability to represent observed extreme temperatures and concluded that the chosen models could not simulate cold days trends. Das et al.^19^ assessed the impact of climate change on temperature extremes and projected a substantial rise in temperature extremes. Wei et al.^20^ evaluated 23 CMIP6 models' ability to simulate extreme climate events over China and stated that considerable uncertainties remain in future climate projections. Although there are indications that GCMs have significant biases in climate simulations compared to observations^21–23^, there have been positive improvements in the CMIP models^24–26^. Hence, selecting an appropriate collection of GCMs is critical for the improved assessment of climate change impacts.

Even though previous researchers focused on the ability of CMIP models to simulate extreme climate events or a particular meteorological variable, none of these studies provides an informed decision on the climate models' skill in representing different sector-specific indices. Neither offers a holistic approach that could be useful for selecting appropriate GCMs when aiming to represent different climate extremes better. It is worth noting that a proper depiction of a global variable's spatial or temporal climatology by a GCM is not the same as the model's ability to represent climate extremes, especially at regional scales, correctly. Additionally, they do not evaluate the multi-index causal effects due to the mutuality of the extreme events. Understanding the causal influence of the candidate's extreme event on the responses of the other extreme occurrences is important.

Therefore, this study focuses on CMIP6 skill in representing multi-index climate extremes for different sectors using eleven CMIP6 models. The sector-specific indices are classified as Health, Agriculture, and Water Resources. We subsequently evaluated the multi-index causal effects for fourteen indices as well as the trends and climate change signals.

## Data and methods

The CMIP6 dataset incorporates different shared socioeconomic pathways (SSPs). This study focuses on eleven CMIP6 models (Supplementary Table S1) under two SSP scenarios (SSP 370 and 585) during historical (1979–2014), near future (2025–2060), and far future (2065–2100) periods for Africa, Asia, North America, South America, Europe, and Oceania. SSP370 is the medium-to-high end of future emissions and temperature scenarios^27^, while SSP585 is the only SSP with emissions sufficient to deliver the 8.5 W/m^2^ level of forcing in 2100. Table 1 presents the sector-based indices^28^.

**Table 1:** List of indices.

S/N	Index	Description (unit)	Name	Sector
1	DTR	The mean difference between the daily maximum temperature and the daily minimum temperature (Celsius)	Daily Temperature Range	AF
2	DTRV	Mean absolute day-to-day variation in the daily temperature range (Celsius)	Daily Temperature Range Variability	AF
3	DSF	The number of dry periods of n days and more, during which the accumulated or maximal daily precipitation amount on a window of n days is under 1.0 mm (days)	Dry Spell Frequency	HW, AF
4	DSTL	Total number of days in dry periods of a minimum length, during which the maximum or accumulated precipitation within a window of the same length is under 1.0 mm (days)	Dry Spell Total Length	HW, AF
5	ETR	The maximum of maximum temperature (TXx) minus the minimum of minimum temperature (TNn) for the given time period (Celsius)	Extreme Temperature Range	AF
6	HWF	The number of heatwaves over a given period. A heatwave is defined as an event where the minimum and maximum daily temperature both exceeds specific thresholds over a minimum number of days, usually for > = 3 days (events)	Heat Wave Frequency	H, HW, AF
7	HWI	Number of days that are part of a heatwave, defined as five or more consecutive days over 27 °C (days)	Heat Wave Index	H, HW, AF
8	HWTL	The total length of heatwaves over a given period. A heatwave is defined as an event where the minimum and maximum daily temperature exceeds 22 °C and 30 °C (respectively) over a minimum number of days. This is the sum of all days in such events (days)	Heat Wave Total Length	H, HW, AF
9	HSF	The number of hot spells over a given period. A hot spell is defined as an event where the maximum daily temperature exceeds 30 °C over a minimum number of days, usually for > = 3 days (events)	Hot Spell Frequency	H, HW, AF
10	DPI	Average precipitation over wet days (mm/day)	Average Daily Precipitation Intensity	HW, AF
11	WDD	The total number of days where warm and dry conditions coincide (days)	Warm and Dry Days	H, HW, AF
12	WSDI	Number of days inside spells of a minimum number of consecutive days where the daily maximum temperature is above the 90th percentile (days)	Warm Spell Duration Index	H, HW, AF
13	HWML	The maximum length of heatwaves over a given period (days)	Heat Wave Maximum Length	H, HW, AF
14	MCCD	Maximum number of consecutive dry days within the period where precipitation is below 1 mm/day (days)	Maximum number of consecutive dry days	H, HW, AF

H, HW and AF denote Health, Hydrology and Water Resources, Agricultural and Food Security, respectively.

The reference dataset, W5E5^29,30^, is a daily-resolution observed climate data on a global 0.5° × 0.5° lat-lon grid. W5E5 was selected as the reference dataset because it has been used to bias-correct climate models for impact studies^31^. All precipitation outputs were re-gridded using second-order conservative mapping due to the significant variability and unequal distribution of precipitation. In contrast, all temperature outputs were re-gridded using bilinear interpolation^32^ to prevent erroneous scale gap effects^31^. All outputs were re-grided to a single 1° × 1° grid.

### GCM ranking

The performance of each GCM was ranked for each climate index during the historical period using five different metrics^23,33–35^; namely, percentage bias, mean absolute error, index of agreement, correlation, and root mean square error. These performance metrics were combined for a universal rank Eqs. (1) and (2) in which all metrics were assigned an equal weight^35,36^.

1 $${T}_{i,j}^{*}=\frac{{T}_{i,j}-{\mathrm{min}(T}_{i,j})}{{\mathrm{max}(T}_{i,j})-{\mathrm{min}(T}_{i,j})}$$

2 $${T}_{i,tot}^{*}=\sum_{j=1}^{m}{T}_{i,j}^{*}$$

where ${T}_{i,j}$ is the error of metric *j* and particular model *i* in space and time. All metrics *m* are added together to get the total relative error ${T}_{i,tot}^{*}$ per GCM per index. It should be noted that the index of agreement and correlation were reversed for the procedures min() and max() because the best-performing models should have a total relative score closer to zero. Rank 1 is the best-performing model for each index.

### Empirical cumulative distribution function (ECDF)

The ECDF is calculated by ordering all the unique observations in the data sample and calculating the cumulative probability for each as the number of observations less than or equal to a given observation divided by the total number of observations (n).

This is given as^37^;

3 $$\widehat{{F}_{n}}(t)=\frac{{\sum }_{i=1}^{n}1{X}_{i}\le t}{n}$$

where 1*X* is the indicator of event *X*. When *t* is fixed, indicator $1{X}_{i}\le t$ is a Bernoulli random variable with parameter p = F(t).

$\widehat{{F}_{n}}(t)$ is the unbiased estimator of F(t). The independent, identically distributed real random variables with the shared cumulative distribution function F(t) are denoted by Xi(t).

We examined the ability of the GCMs to simulate the monthly climatology of the extreme indices by visually inspecting and subjecting the ECDF distributions to Kolmogorov–Smirnov test to understand the distribution differences between the reference and the outputs from the GCMs.

### Correlation, partial correlation, index of importance and causal effects

The degree of association is a metric that shows how strong the relation between two variables is without considering that a third variable may influence both variables. When several factors impact the phenomenon under examination, partial correlation becomes extremely important.

However, due to the changing variance and linearity of the extreme events, we used the spearman correlation to benchmark the monotonic relationship between the extreme events.

Partial correlation is the correlation of two variables while controlling for a third or more other variables. The relationship is said to be partial when two variables are correlated while conditioning the third or several other variables^38^.

The partial correlation is given as:

4 $$\rho UVW= \frac{\rho UV- \rho UW\rho VW}{\sqrt{\left(1-{\rho }^{2}UW\right)*(1-{\rho }^{2}VW)}}$$

where $\rho UVW$ is the partial correlation of variables *U* and *V* conditioned on *W*, $\rho UV$ is the correlation between varaibles *U* and *V*, $\rho UW$ is the correlation between variables *U* and *W* while $\rho VW$ is the correlation between variables *V* and *W.*

For a set of *t* controlled variables, *W* is

$$W= \left\{{W}_{1},{W}_{2}, .., {W}_{t}\right\}$$

Statistically significant correlation and partial correlation were examined using the Student's t-test at a 95%, 99% and 99.9% confidence level.

Due to the mutuality of these extreme events, we also quantified the causal influence of the candidate's extreme event on the responses of the other extreme occurrences.

Several methods have been proposed to assess this, but the most popular is the permutation of importance, based on the random forest algorithm^39,40^. However, this approach is unsuitable for highly correlated events, as it cannot distinguish between an event's conditional and marginal influence^41^.

As a result, the Conditional Variable Relevance—also known as the Conditional Permutation Importance (CPI)—was used to assess more partial importance in random forests. The CPI is presented as:

- For the regression trees in the random forest
  5 $${R}^{(t)}=\sum_{{i\in \beta }^{(t)}}\frac{{\left({\widehat{y}}_{i}^{(t)}-{y}_{i}\right)}^{2}}{\left|{\beta }^{(t)}\right|}$$
  6 $${R}_{(k)}^{(t)}=\sum_{{i\in \beta }^{(t)}}\frac{{\left({\widehat{y}}_{i(k)}^{(t)}-{y}_{i}\right)}^{2}}{\left|{\beta }^{(t)}\right|}$$
- For the classification trees in the random forest
  7 $${R}^{(t)}=\sum_{{i\in \beta }^{(t)}}\frac{I\left({\widehat{y}}_{i}^{(t)}\ne {y}_{i}\right)}{\left|{\beta }^{(t)}\right|}$$
  8 $${R}_{(k)}^{(t)}=\sum_{{i\in \beta }^{(t)}}\frac{I\left({\widehat{y}}_{i(k)}^{(t)}\ne {y}_{i}\right)}{\left|{\beta }^{(t)}\right|}$$
  $${\widehat{y}}_{i(k)}^{(t)}={f}^{(t)}{(x}_{i(k)})$$
  $${x}_{i(k)}={(x}_{i}1, \dots ,{x}_{ik-1, }{x}_{p\left(i\right)k, }{x}_{ik+1}, \dots , {x}_{ip})$$

where ${R}^{(t)}$ and ${R}_{(k)}^{(t)}$ are the prediction error of tree *t* in a random forest with predictors *p* and *ntrees* of trees based on the out-of-bag sample ${\beta }^{(t)}$, respectively, before the permutation of the out-of-bag values of ${X}_{k}$. $\left|{\beta }^{(t)}\right|$ is the out-of-bag cardinal number sample for tree *t*, and *I*() is the indicator function.

${\widehat{y}}_{i}^{(t)}$ is the function ${f}^{(t)}{(x}_{i})$, which is the random forest prediction of the out-of-bag observation *i* before permutation. ${x}_{p\left(i\right)k}$ is the *i*th ${X}_{k}$ observation after permutation.

The forest permutation of importance ${PI}_{(k)}$ is the average overall tree-wise permutation ${PI}_{(k)}^{(t)}$.

$${PI}_{(k)}=\frac{\sum_{t=1}^{ntree}{PI}_{(k)}^{(t)}}{ntree}$$

where $${PI}_{(k)}^{(t)}$$= $${R}_{(k)}^{(t)}= {R}^{(t)}$$

In the CPI, out-of-bag values of one predictor ${X}_{(k)}$ are conditionally permuted on other predictors ${Z}_{(-k)}$.

The extreme events are subjected to CPI following Debeer and Strobl^40^, while the causality assumptions are presented by van der Laan^42^.

### Climate change signals in extreme events

For risk management purposes, climate projections should include a wide range of feasible spectrums of anticipated future climate change^36^. The climate change signals (CCS) in extreme events were calculated as the difference between (i) the far future and the historical period, (ii) the near future and the historical period, and (iii) the far future and the near future.

### Trends and significance

Spatial plots based on robustness and significance criteria are presented for all indices' historical and future assessments. Following Haensler et al.^43^, this study considers a signal robust if 66% of the models agree. Statistically significant trends and climate change signals were examined using the Student's t-test at a 95% confidence level^35^. The trend in the climate extreme events was calculated based on the Mann–Kendall trend test, while the trend's magnitude was estimated using Sen's slope^35,44–46^.

## Results

### Spatial spread climatology

**Figure 1:**
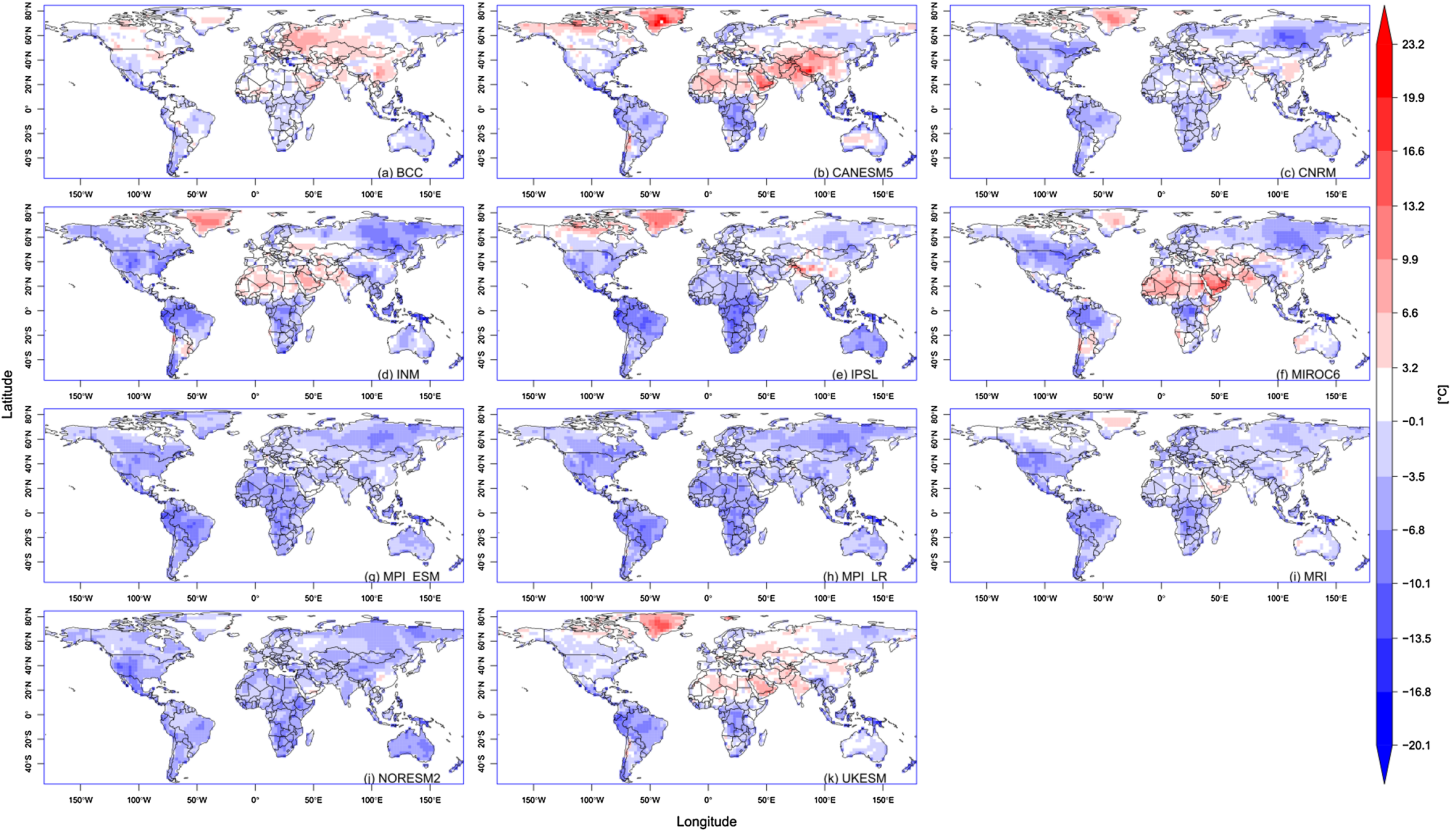
Bias in the monthly climatology of ETR for different CMIP6 models.

The climatology was computed for all indices for the historical period. For instance, the climatology of extreme temperature range (ETR) ranged from 2 to 47 °C (Supplementary Fig. S1), with the tropics having the smallest range. BCC replicated the spatial spread, with slight overestimation over Russia and underestimation around the Brazilian and West African coasts. Additionally, other models had varying degrees of overestimation or underestimation. Generally, the range increased away from the equator in the Northern and Southern Hemispheres. This pattern is visible in all models. The departure of the models from the reference is presented in Fig. 1. Most models had evident cold biases in the Southern Hemisphere. However, some models, e.g., BCC, performed well in North America, Greenland, West Africa, and some parts of Russia. The hot bias was highest in CANESM5, with ETR values as high as 23 °C in Greenland and China. MPI-ESM, MPI-LR, and NORESM2 all recorded cold tendencies over entire continents. The spatial spread for other indices is presented in the supplementary file (Supplementary Fig. S2 to S9).

### Regional ECDF

Taking the ETR as an example, Supplementary Fig. S10 shows the distribution of the ECDF on the continental scale. Over Africa, four models (CANESM5, BCC, UKESM, and INM) reasonably replicated the reference ETR (Supplementary Fig. 10a, Table 2). However, based on the Kolmogorov–Smirnov statistics (Table 2), INM showed the lowest distribution distance (0.14), although with a P-value lesser than 0.05. This means INM performed best in capturing the ECDF distribution. However, the P-value suggested that INM distribution was not the same as the reference distribution. MIROC6 overestimated this by up to 5 °C. At the same time, IPSL, MPI-LR, MPI-ESM, and NORESM2 underestimated the distribution by up to 7 °C. Over Asia, there was no outright distinction in the distribution by the models. Nonetheless, MIROC6 was close to the reference with a Kolmogorov–Smirnov statistic of 0.15. Over South America, no model could replicate the reference distribution well, although BCC performed better than the other models. The Kolmogorov–Smirnov statistics is 0.48. All models behaved differently in capturing the ECDF of different indices and for separate continents.

**Table 2:** Kolmogorov–Smirnov statistics for ECDF comparison.

Continents		BCC	CANESM5	CNRM	INM	IPSL	MIROC6	MPI_ESM	MPI_LR	MRI	NORESM2	UKESM
Africa	Statistics	0.33	0.37	0.45	**0.14**	0.53	0.32	0.45	0.57	0.7	0.59	0.17
	P-value	4.50E−06	2.00E−02	1.00E−18	8.40E−11	1.00E−19	1.00E−E−20	1.00E−20	1.00E−17	1.00E−20	1.00E−14	2.00E−04
Asia	Statistics	0.25	0.43	0.27	0.35	0.34	**0.2**	0.5	0.56	0.37	0.62	**0.2**
	P-value	2.90E−12	1.00E−19	1.60E−14	1.00E−20	1.00E−20	7.98E−08	1.00E−17	1.00E−20	1.00E−14	1.00E−19	7.90E−08
North America	Statistics	0.19	0.3	0.31	0.17	0.4	**0.15**	0.47	0.51	0.35	0.52	0.25
	P-value	2.51E−07	1.00E−14	1.00E−19	4.48E−06	1.00E−19	6.00E−05	1.00E−19	1.00E−20	1.00E−17	1.00E−20	2.92E−12
South America	Statistics	**0.48**	0.70	0.72	0.74	0.98	0.61	0.99	0.99	0.95	0.94	0.99
	P-value	1.00E−19	1.60E−14	1.00E−20	1.00E−20	1.00E−20	1.00E−17	1.00E−20	1.60E−14	1.00E−20	1.00E−20	1.00E−20
Europe	Statistics	0.33	0.36	0.45	**0.14**	0.53	0.32	0.45	0.57	0.70	0.59	0.17
	P-value	1.00E−17	1.00E−20	1.00E−14	1.00E−19	1.00E−19	1.00E−20	1.00E−20	1.00E−17	1.00E−14	1.00E−19	1.00E−17
Oceania	Statistics	0.48	0.39	0.65	0.57	0.94	**0.26**	0.69	0.69	0.30	0.94	0.37
	P-value	1.00E−20	1.00E−20	1.00E−20	1.00E−17	1.00E−19	1.38E−13	1.00E−19	1.00E−20	1.00E−20	1.00E−17	1.00E−20

Bold values represent the lowest distance (high similarity to reference). High P-values show that the reference and CMIP6 model distribution are identical.

### GCM performance and ranking

**Figure 2:**
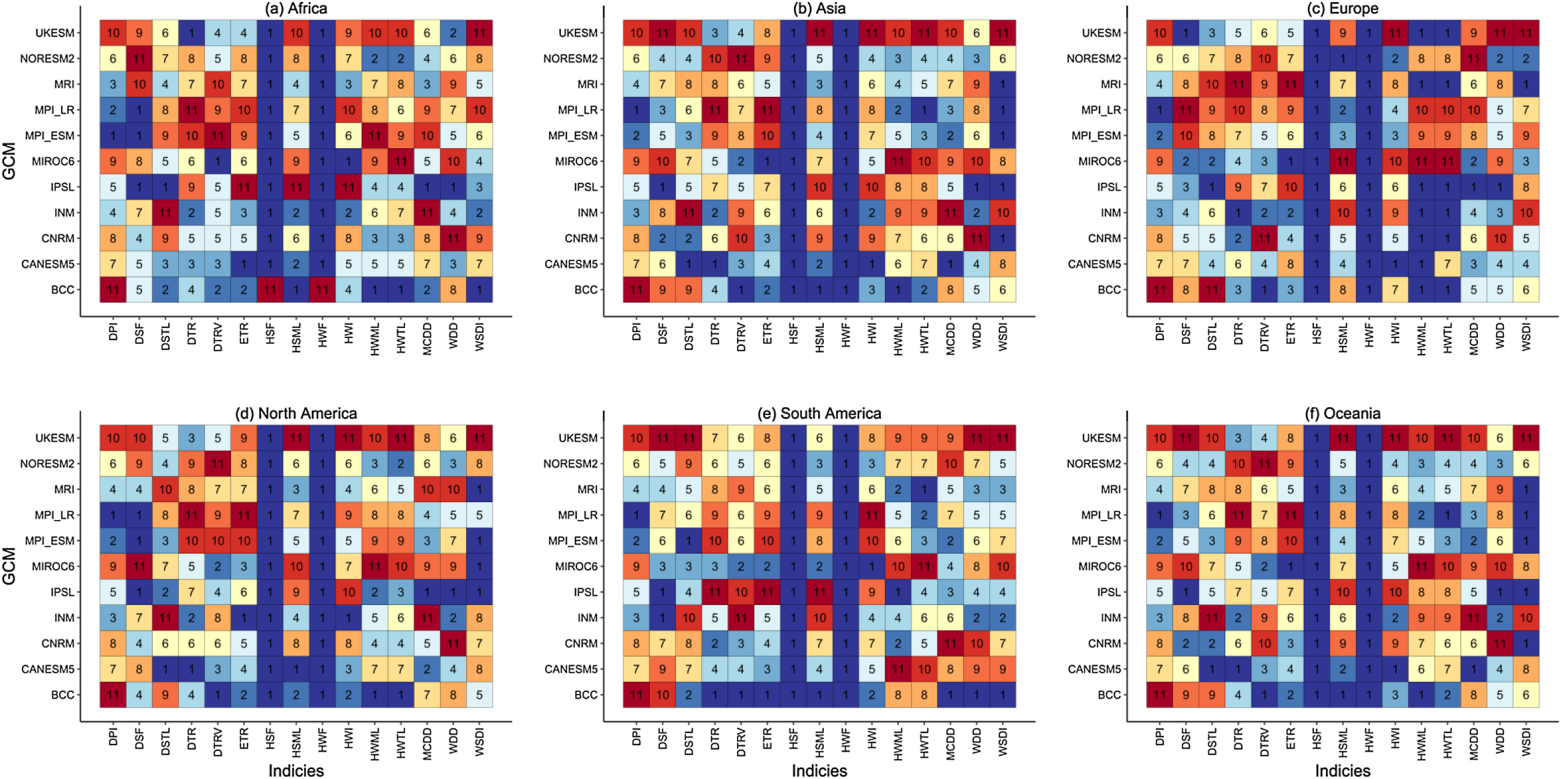
Multi-index continental GCM ranks.

Figure 2 shows the multi-index continental GCM ranks. Over Africa (Fig. 2a; Supplementary Table S2), MPI-ESM performed best in replicating the daily precipitation intensity (DPI), while BCC performed worst (ranked 11th). For ETR, four models (CANESM5, BCC, UKESM, and INM), as observed in the ECDF earlier, ranked best in Africa. Over Asia, CANESM5 ranked first in heatwave index (HWI), maximum consecutive dry days (MCCD), daily temperature range (DTR), and dry spell total length (DSTL). In contrast, BCC ranked first in heatwave maximum length (HWML), hot spell maximum length (HSML), and DTRV. As observed by the ECDF of ETR over Asia, MIROC6 was the best-performing model in ETR. Over Europe and North America, IPSL ranked first in replicating the observed warm and dry days (WDD). In contrast, MPI-LR ranked first in reproducing the reference DPI over South America and Oceania, respectively. Notably, the hot spell frequency (HSF) and heat wave frequency (HWF) were well replicated by all CMIP6 models, as the error margin was between 2 to − 3 days/decade for all models.

### Correlation and partial correlation

**Figure 3:**
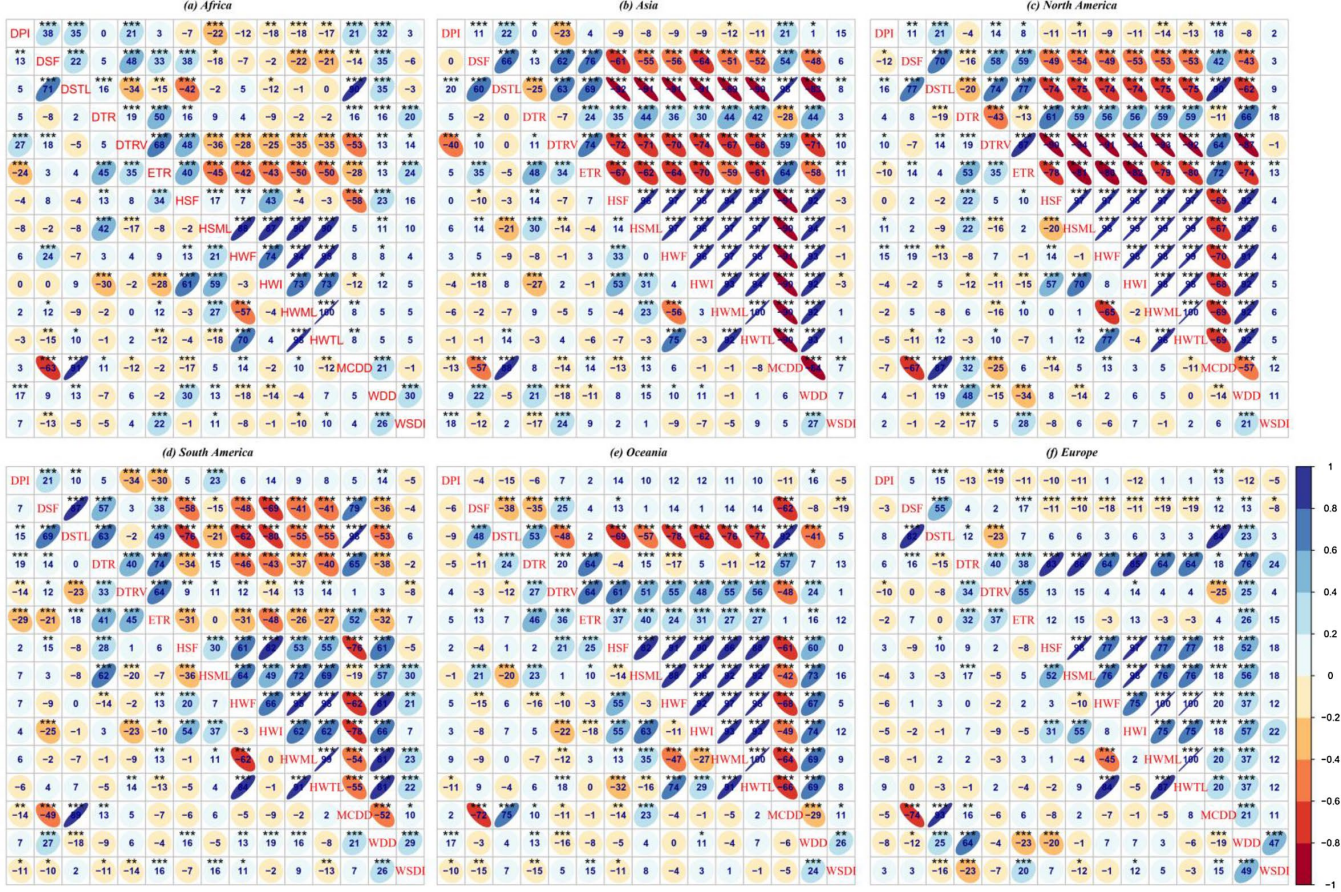
Multi-index correlation and partial correlation plot for the reference over (**a**) Africa (**b**) Asia (**c**) North America (**d**) South America (**e**) Oceania (**f**) Europe. The upper diagonal to the indices is the correlation values, lower diagonal to the indices is the partial correlation values. *, ** and *** represents significant correlation, and partial correlations at P-value = 0.001, 0.01 and 0.05 respectively.

Figure 3 shows the multi-index correlation and partial correlation plot for the reference on the continental scale. For instance, in Africa, the HSML was strongly positively correlated with heatwave frequency (HWF) (88%), HWI (87%), HWML (90%), and heatwave total length (HWTL) (90%), all at the 95% confidence level (upper diagonal). However, this is different when considering partial correlation. The partial correlation plot (lower diagonal) shows that the HSML was positively correlated with heatwave frequency (HWF) (21%), HWI (59%), and HWML (27%), while negatively correlated with HWTL (− 18%), all at the 95% confidence level. The same pattern was observed in Asia and Oceania. The partial correlation values and directions differed in North America, South America, and Europe. The analysis was repeated for the ensemble-mean of the CMIP6 (Supplementary Fig. S11). Generally, the reference and model ensemble-mean have similar correlation and partial correlation patterns, albeit with differing magnitudes.

### Causality and importance

**Figure 4:**
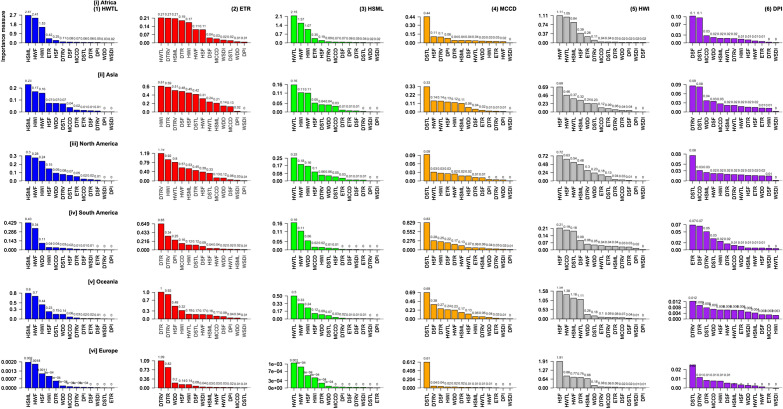
Conditional variable of importance of selected extreme indices using the reference.

Figure 4 presents the CPI results for the reference data. Over the entire continent, HWTL was greatly influenced by the hot spell maximum length (HSML) and HWF. There was no universal pattern of causality for ETR, as the importance-modulating index varied with the continent. In Africa, HWTL, DTRV, and HSML were the most influential indices for ETR, while in Asia, HWI, DTR, and DTRV were the most significant. In contrast, DTRV and DTR were the most influential indices in Europe. HWTL and HWF greatly influenced changes in the pattern of HSML on all continents, while DSTL was the most crucial index modulating the MCCD for all continents. Supplementary Fig. S12 to S18 show the CPI of other extreme indices over the entire continent. Additionally, there was general agreement between the index of importance observed in the reference and the multimodel ensemble-mean.

### Trends and CMIP6 trend consistency

**Figure 5:**
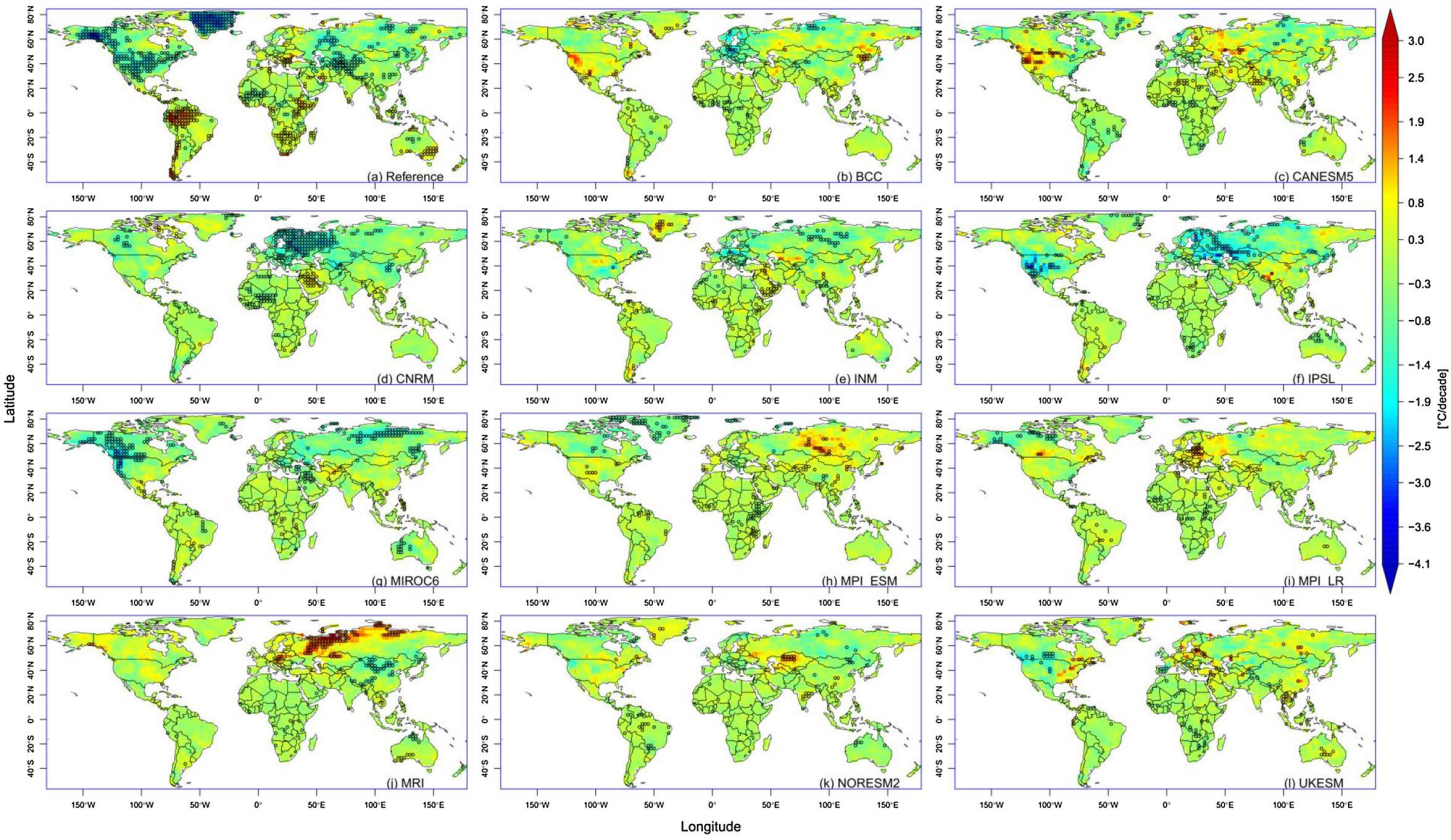
ETR trend for CMIP6 models during the historical period. Hatchings represent area with significant trend at 95% confidence level.

As a case study, Fig. 5 shows the trends in ETR for reference and the CMIP6 models during the historical period. For reference, there were some statistically significant negative trends in ETR in some parts of North America and Asia, with values between − 4.1 and − 0.3 °C/decade. However, there was a typical positive trend of ETR in Africa and South America, though some parts were not statistically significant. Moreover, the CMIP6 models could not capture the significant trends in the reference. They also exhibited some underestimations, particularly in South America, and overestimations in Greenland. Except for IPSL and CNRM, most models showed a general warm bias over North America and northern Asia (Supplementary Fig. S19). Furthermore, there was a characteristic cold bias in South America, Africa, and Oceania. The range of the model trend biases was between − 3.4 and 4.7 °C/decade.

**Figure 6:**
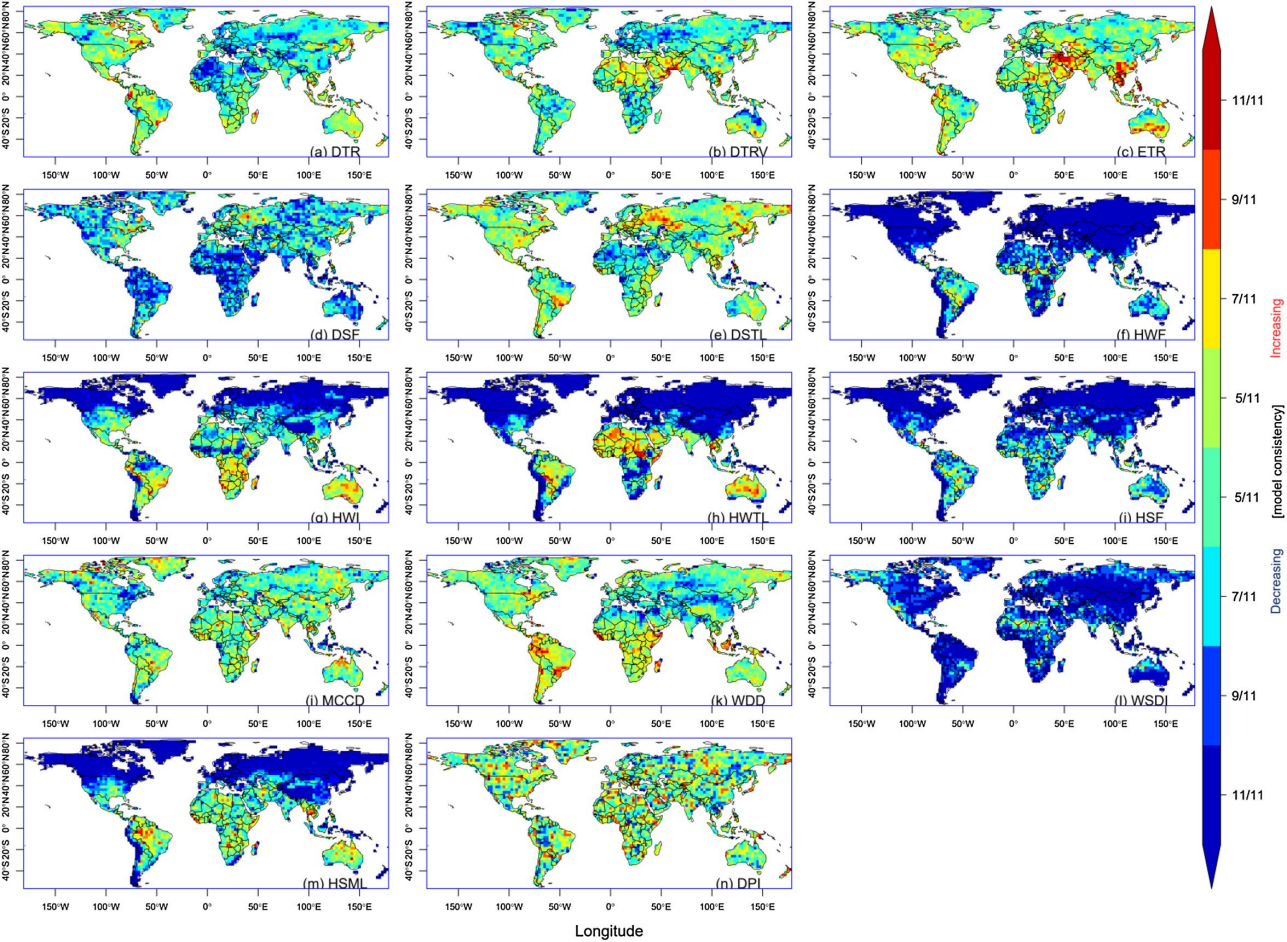
Trend consistency for CMIP6 models during the historical period.

There was a consistent negative trend of DTR over most parts of West and North Africa, southern Europe, and Central Asia, with agreement by all eleven models (Fig. 6). The positive DTRV trend consistency was prominent mainly between latitudes 15° N and 35° N; and longitudes 25° W and 60° E, while an apparent negative trend was observed over Europe, northern Australia, and western Canada, with agreement by at least nine models. The consistent positive trend of HTWL was particularly noticeable in West and North Africa, most parts of Australia, and South America.

### Projection and change signal

**Figure 7:**
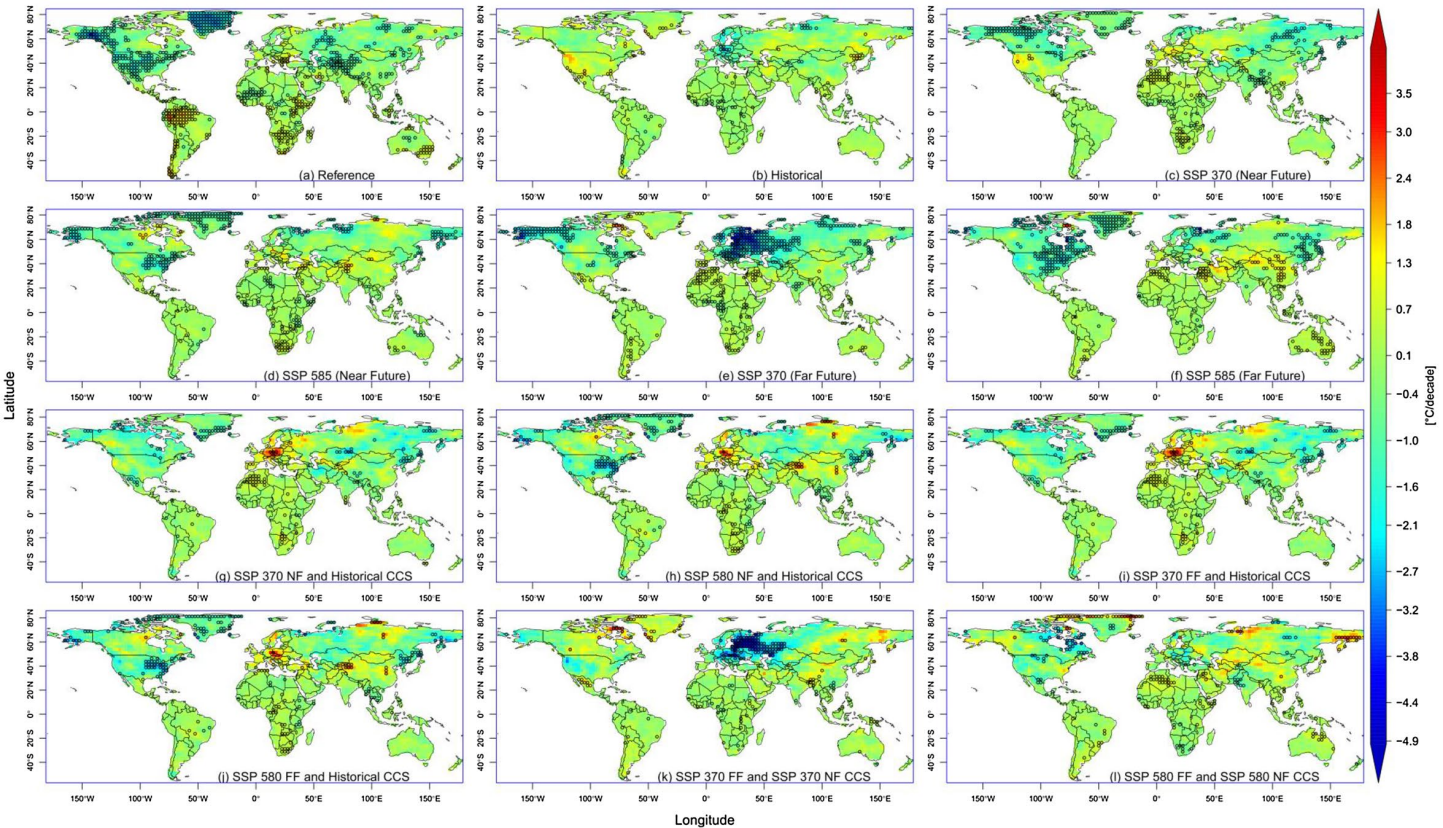
Trends of ETR during the reference (**a**), historical and future periods (**b**–**f**), climate change signals (CCS) in trends between the historical, near future (NF) and far future (FF) (**g**–**l**) for both SSP 370 and 585 using CMIP6 ensemble mean. Hatchings represent area with significant trend at 95% confidence level.

Taking ETR as a case study, Fig. 7 shows the CCS in trends between the historical, near future (NF), and far future (FF) for both SSP 370 and 585 using the CMIP6 ensemble-mean. Although there were some significant negative trends of ETR in some parts of North America and some significant positive trends in some parts of South America, the ensemble-mean generally replicated the spatial trend in other parts of the world during the historical period, although with different magnitudes and significance. In most cases, most parts of the tropics witnessed a positive trend of ETR during the historical period. The FF under SSP 370 witnessed a significant widespread negative trend in Europe and Alaska. Similarly, other parts of the world mainly had positive trends. The trend values varied between − 4.9 and 3.5 °C/decade.

We further explored the climate change signal in the trends for the different periods.

The CCS between the historical and SSP 370 NF showed a rise in the trend of ETR from the historical to SSP 370 NF periods in Europe, with a magnitude between 1.0 and 4.0 °C/decade. However, there was a general drop in this trend for most parts of Canada and some parts of Russia. Moreover, most parts of the world experienced an increasing trend from the historical to SSP 370 NF. In reverse, the CCS between the historical and SSP 585 NF revealed that most areas of Canada that were previously accustomed to negative trends were reversed to positive. In contrast, the once positive trend in the USA under SSP 370 NF became negative. China also had a more intense positive trend during this period. A further look into the CCS between SSP 370 NF and SSP 370 FF shows a significant deepening in the trend of ETR over most parts of Europe, with magnitudes of between − 2.1 and − 5.0 °C/decade. At the same time, some other areas like north-eastern Canada had a significant rise in the trend by up to 4 °C. Supplementary Fig. S20 shows the result of the same experiment considering DTR.

**Figure 8:**
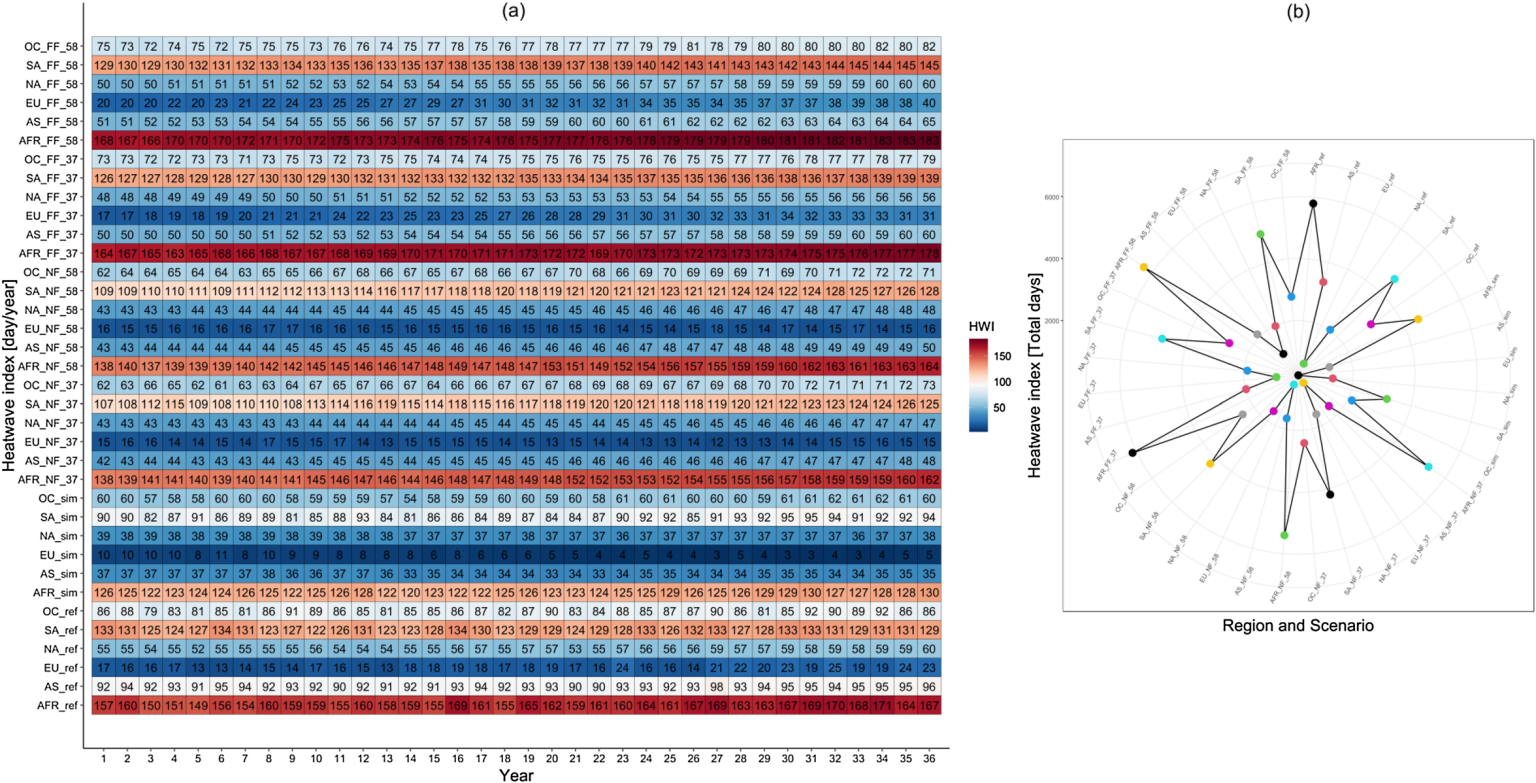
Yearly cumulative heatwave index for the reference (ref), historical (sim), near future (NF) and far future (FF) (**b**) Total cumulative heatwave index. AFR, AS, EU, NA, SA and OC mean Africa, Asia, Europe, North America, South America, and Oceania, respectively. 37 and 58 denote SSP 370 and 585, respectively. On the year axis, the first index represents the starting year of each period, i.e., 1979, 2025 and 2065, for historical, Near future and far future periods, respectively. The box's border in Fig. 8b indicates the circumference axis, with the smallest circumference starting with 2000.

Figure 8 shows the yearly and total cumulative heatwave index (HWI). Africa had the highest days of HWI. The reference series values ranged from 149 (in 1983) to 171 days/year (in 2012). However, the highest days in the historical CMIP6 ensemble-mean varied from 120 to 130 days/year. The highest days of HWI for the entire period of study manifested during the far future under SSP 585, with 181 days/year from 2098 to 2100. Nevertheless, comparing the reference with the historical simulations showed an underestimation of HWI days for all continents. The total cumulative HWI index showed Africa having more than 6000 days of HWI during the far future under SSP 370 and 585, while Asia maintained a value of 2000 days under the same SSPs.

### Zonal and meridional cross-section ETR

**Figure 9:**
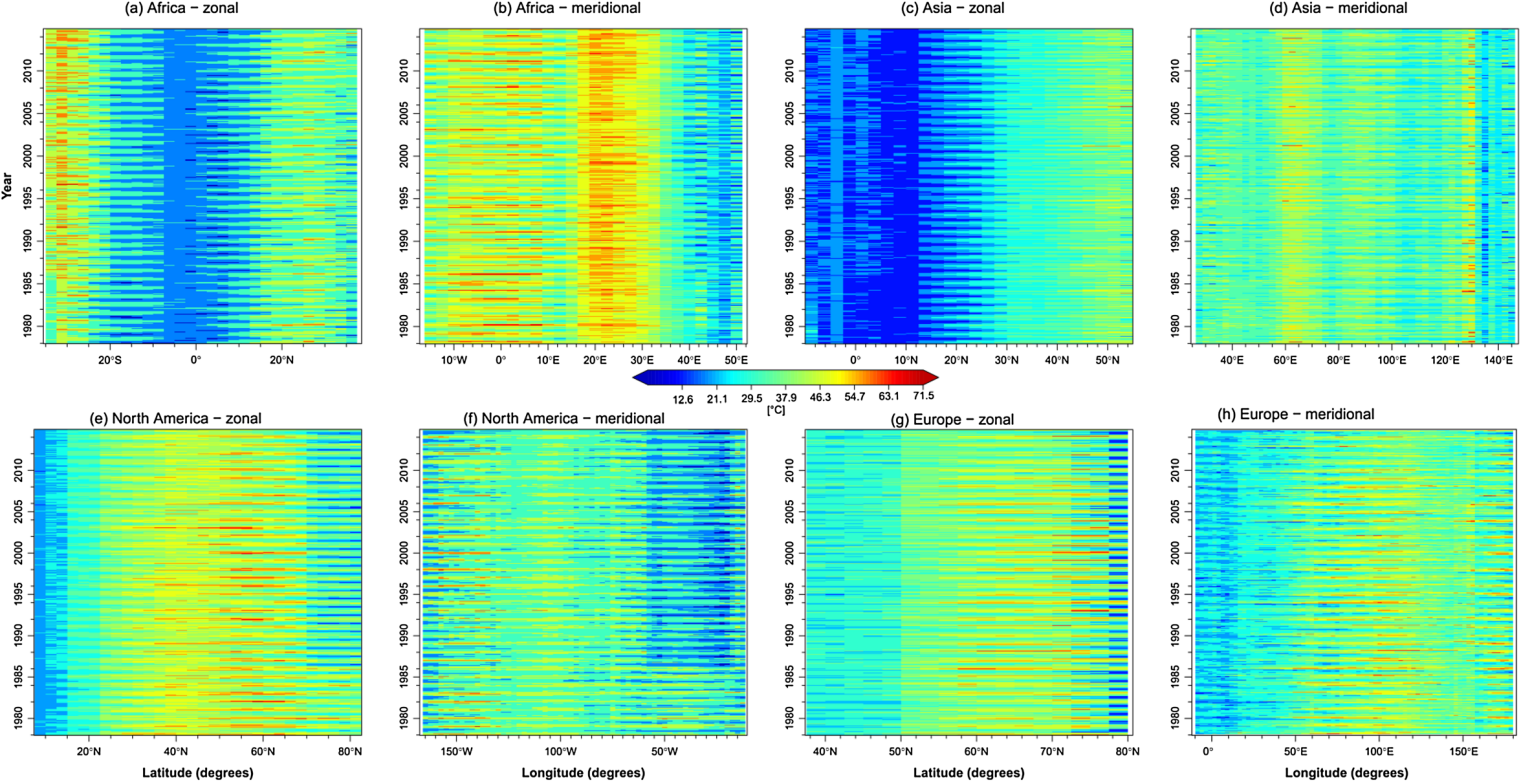
Zonal and meridional cross-section of reference ETR.

Zonal and meridional cross-sections were used to understand further the influence of land, sea, and latitude differences on ETR (Fig. 9). Over Africa, there was an apparent increase of ETR away from the equator and the coast. However, high ETR was visible in desert regions, specifically between 20 and 30°N (Sahara Desert) and between 25 and 32°S (Kalahari Desert). More importantly, the ETR was highest in the Kalahari Desert for all years due to cooler (freezing) nights than in the Sahara Desert. Furthermore, the ETR decreased toward the Mediterranean due to the ocean's high specific heat capacity. ETR was highest in regions with a dense landmass and reduced toward the ocean due to the land's low specific heat capacity compared to the ocean.

ETR was lowest over scattered landscapes (− 10° N to 10° N) in Asia and increased with denser landmass (Fig. 9c). Meridionally, ETR was highest in mountainous regions due to the ability of these regions to warm and cool faster than the surrounding regions. ETR also decreased toward the Pacific Ocean. Generally, ETR ranged between 3.0 and 72.0 °C for the displayed continents.

**Figure 10:**
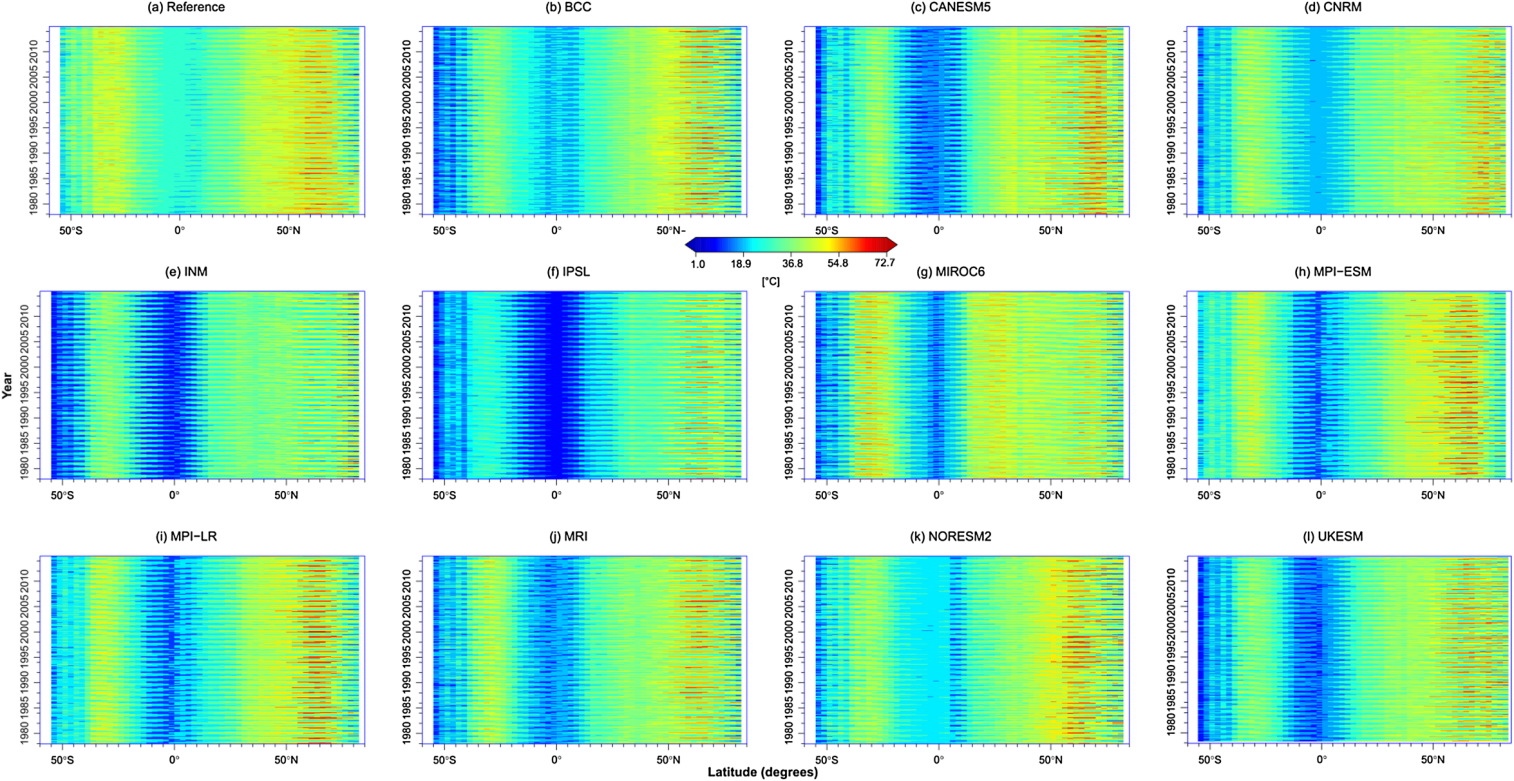
Zonal cross-section of the reference and CMIP6 ETR during the historical period.

Additionally, there were cold biases in most CMIP6 models along the tropics (Fig. 10). However, NORESM2 reasonably captured the low ETR along the equator and a few degrees away from the equator. IPSL and INM underestimated the low ETR along the equator by 5 to 20 °C. In contrast, MPI-LR, MPI-ESM, CANESM5, and BCC captured the high ETR north of 50°N reasonably well. The models, overall, mirrored the reference patterns. The ETR reduced as it approached the Arctic due to ice, increased as it advanced toward the Mediterranean, and declined as it approached the equator. The broad range was between 1.0 and 73.0 °C.

## Discussion and conclusion

In this study, we explored the potentials of eleven GCMs participating in CMIP6 project in representing fourteen climate extreme indices that are useful for different sectors, including health, agriculture and water resources. Understanding of climate model skills, multi-index causal effects and global climate change signals of extremes is limited, but is key in reinforcing well-informed decisions in the various sectors.

For the selected models, individual CMIP6 GCM results often varied significantly between regions and models. For example, the top-performing GCM in simulating the reference DPI tended to exhibit consistently strong performance across continents, whereas the best-performing model in simulating DTSL differed among continents.

Additionally, there were observable biases in the climatology of various indices. The distribution of the ECDF supported these inconsistencies. Notably, all models behaved differently in capturing the ECDF of different indices and for different continents. These inconsistencies could be attributed to the various land surface schemes and simulation of features such as vegetation^36^ and orography^47^, unrealistic large-scale variability^35,48^, and contrasting internal variability between climate models and observations^23,49^.

Nevertheless, previous studies^50–52^ showed that CMIP GCMs struggled to capture daily temperature minima and maxima. Yet, understanding extreme temperature events is critical since many earth system processes are influenced by the maximum and lowest temperatures as well as the mean temperature^36,52^.

The performance of GCM in simulating precipitation extremes also varied widely across the globe. However, it is essential to note that the highest biases were recorded immediately north and south of the equator, particularly for DSTL. The signal of these biases varied among the different models, suggesting the inability of the models to correctly resolve important biophysical features such as solar radiation, vegetation, and cloud features, especially at daily scales^53,54^ or other synoptic-scale system interactions^55^. In contrast to Srivastava et al.^56^, who reported that UKESM was one of the best-performing models in the US for precipitation simulation, we found that IPSL performed best in simulating MCCD for both Africa and North America, while MPI-ESM and MPI-LR performed best in simulating DPI in Africa and North America, respectively. Across all continents, IPSL performed best for MCCD, while MPI-LR performed best for DPI. Remarkably, the MPI model family performed best in capturing the DPI across the six continents. This may have been produced from model inter-dependence^36,56,57^.

Despite the different biases associated with the various models, the correlation and partial correlation patterns were similar for the reference and model ensemble-mean, although with different magnitudes. Also, the causal impact for different indices varied. More precisely, the causal impact for ETR did not follow a universal pattern since the important modulating index differed depending on the continent. Furthermore, the multimodel ensemble-mean and the significant index agreed with the reference.

There was a mix of trends for the various specific indices for each location. Despite a rise in both maximum and minimum temperatures, the significant negative trends of DTR suggested quicker warming of the minimum temperature^2^.

Nonetheless, these results have some practical implications for decision-makers in different sectors. Due to changes in land cover, land use, and the resources employed in urban centres, cities have greater surface temperatures than rural areas (urban heat island phenomenon^53,58–60^). Additionally, urban heat island impacts lengthen the duration of heat events during heatwaves^61^; however, this behaviour is different for the various atmospheric circulation regions. For example, we could not observe increased heatwave total length in most urban areas in the mid-latitudes. Yet, the reverse is for urban areas in the tropics. This demonstrates that rather than urban heat islands, large-scale meteorological conditions^62^, significantly impact the length of the duration of heatwaves in midlatitude.

The consequences of drought on water demand and supply by natural systems and people, on the other hand, will be amplified as the climate warms^63,64^. Furthermore, rising temperatures worsen heavy precipitation by increasing atmospheric moisture, promoting the precipitation event through moisture convergence at low altitudes^65,66^ and increasing evapotranspiration rates arid areas^46^. This will lead to more intense hydrometeorological situations, such as floods and droughts^5^ and significantly impact the amount and quality of available water as well as river discharge timing and amplitude^67^. As a result, humans, society and natural systems are at risk^67,68^.

Therefore, it is crucial to design appropriate strategies to strengthen resilience to extreme climatic events while also mitigating further GHG emissions. However, optimal adaptation/mitigation strategies for climate change could be hampered by incorrect information from the poor representation of climatic events in data sets.

Beyond the specifics of this research, future work could investigate extreme historical events with additional reference datasets, bias-corrected or downscaled CMIP6 datasets, especially for extreme precipitation events. Additionally, more CMIP6 models could be utilized.

## Supplementary Information

Supplementary Information.

## Data Availability

CMIP6 data are publicly available through the Earth System Grid Federation at: http://esgf.llnl.gov/. The W5E5 reference dataset is distributed by the GFZ Data Services and can be downloaded at https://doi.org/10.5880/pik.2019.023. The derived data generated for the study are available from the corresponding author on reasonable request.

## Code availability

All analyses and figure were drawn in the R version 4.1.2 Platform^69^ (https://www.R-project.org) and Python version 3.9^70^ (https://www.python.org/).

## References

[1] Hassan Z, Shamsudin S, Harun S (2014). Application of SDSM and LARS-WG for simulating and downscaling of rainfall and temperature. Theor. Appl. Climatol..

[2] Adeyeri OE, Lawin AE, Laux P, Ishola KA, Ige SO (2019). Analysis of climate extreme indices over the Komadugu–Yobe basin, Lake Chad region: Past and future occurrences. Wea. Clim. Extrem..

[3] Climate and Environment. Iceberg Splits From Antarctica, Becoming World’s Largest. *The New York Times* (2021).

[4] Luo X, Keenan TF (2022). Tropical extreme droughts drive long-term increase in atmospheric CO2 growth rate variability. Nat. Commun..

[5] Pal SC (2022). Threats of climate change and land use patterns enhance the susceptibility of future floods in India. J. Environ. Manage..

[6] Orlowsky B, Seneviratne SI (2013). Elusive drought: Uncertainty in observed trends and short- and long-term CMIP5 projections. Hydrol. Earth Syst. Sci..

[7] Seneviratne SI, Field CB, Barros V, Stocker TF, Dahe Q (2012). Managing the Risks of Extreme Events and Disasters to Advance Climate Change Adaptation.

[8] Seneviratne SI, Hauser M (2020). Regional climate sensitivity of climate extremes in CMIP6 versus CMIP5 multimodel ensembles. Earth's Fut..

[9] Eyring V (2016). Overview of the coupled model intercomparison project phase 6 (CMIP6) experimental design and organization. Geosci. Model Dev..

[10] Bai H (2021). Multi-model ensemble of CMIP6 projections for future extreme climate stress on wheat in the North China plain. Int. J. Climatol..

[11] Bourdeau-Goulet S-C, Hassanzadeh E (2021). Comparisons between CMIP5 and CMIP6 models: Simulations of climate indices influencing food security, infrastructure resilience, and human health in Canada. Earth's Fut..

[12] Chen H, Sun J, Lin W, Xu H (2020). Comparison of CMIP6 and CMIP5 models in simulating climate extremes. Sci. Bull..

[13] Ge F, Zhu S, Luo H, Zhi X, Wang H (2021). Future changes in precipitation extremes over Southeast Asia: Insights from CMIP6 multi-model ensemble. Environ. Res. Lett..

[14] Narsey SY (2020). Climate change projections for the Australian monsoon from CMIP6 models. Geophys. Res. Lett..

[15] Chen C-A, Hsu H-H, Liang H-C (2021). Evaluation and comparison of CMIP6 and CMIP5 model performance in simulating the seasonal extreme precipitation in the Western North Pacific and East Asia. Wea. Clim. Extrem..

[16] Ridder NN, Ukkola AM, Pitman AJ, Perkins-Kirkpatrick SE (2022). Increased occurrence of high impact compound events under climate change. NPJ Clim. Atmos. Sci.

[17] Dike VN, Lin Z, Fei K, Langendijk GS, Nath D (2022). Evaluation and multimodel projection of seasonal precipitation extremes over central Asia based on CMIP6 simulations. Int. J. Climatol..

[18] Collazo S, Barrucand M, Rusticucci M (2022). Evaluation of CMIP6 models in the representation of observed extreme temperature indices trends in South America. Clim. Change.

[19] Das S, Islam ARMT, Kamruzzaman M (2022). Assessment of climate change impact on temperature extremes in a tropical region with the climate projections from CMIP6 model. Clim. Dyn..

[20] Wei L (2022). Simulation and projection of climate extremes in China by multiple coupled model intercomparison project phase 6 models. Int. J. Climatol..

[21] Laux P (2021). To bias correct or not to bias correct? An agricultural impact modelers’ perspective on regional climate model data. Agric. For. Meteorol..

[22] Trenberth KE (2011). Changes in precipitation with climate change. Clim. Res..

[23] Adeyeri OE, Laux P, Lawin AE, Oyekan KSA (2020). Multiple bias-correction of dynamically downscaled CMIP5 climate models temperature projection: A case study of the transboundary Komadugu-Yobe river basin, Lake Chad region, West Africa. SN Appl. Sci..

[24] Li C (2021). Changes in annual extremes of daily temperature and precipitation in CMIP6 models. J. Clim..

[25] Xu Y, Zhang X, Hao Z, Hao F, Li C (2021). Projections of future meteorological droughts in China under CMIP6 from a three-dimensional perspective. Agric. Water Manag..

[26] Yao N (2020). Projections of drought characteristics in China based on a standardized precipitation and evapotranspiration index and multiple GCMs. Sci. Total Environ..

[27] O'Neill BC (2016). The scenario model intercomparison project (ScenarioMIP) for CMIP6. Geosci. Model Dev..

[28] Logan, T. *et al. Ouranosinc/xclim: v0.35.0* (Zenodo, 2022).

[29] Cucchi M (2020). WFDE5: Bias-adjusted ERA5 reanalysis data for impact studies. Earth Syst. Sci. Data.

[30] Weedon GP (2014). The WFDEI meteorological forcing data set: WATCH forcing data methodology applied to ERA-Interim reanalysis data. Water Resour. Res..

[31] Iturbide M (2022). On the need of bias adjustment for more plausible climate change projections of extreme heat. Atmos. Sci. Lett..

[32] Agrafiotis D, Bull DR (2014). Academic Press Library in Signal Processing.

[33] Gómez-Navarro JJ, Montávez JP, Jerez S, Jiménez-Guerrero P, Zorita E (2012). What is the role of the observational dataset in the evaluation and scoring of climate models?. Geophys. Res. Lett..

[34] Xuan W (2017). Evaluating historical simulations of CMIP5 GCMs for key climatic variables in Zhejiang Province, China. Theor. Appl. Climatol..

[35] Dieng D (2022). Multivariate bias-correction of high-resolution regional climate change simulations for West Africa: Performance and climate change implications. JGR Atmos..

[36] Di Virgilio G (2022). Selecting CMIP6 GCMs for CORDEX dynamical downscaling: model performance, independence, and climate change signals. Earth's Fut..

[37] van der Vaart AW (2012). Asymptotic Statistics.

[38] Kim S (2015). ppcor: An R package for a fast calculation to semi-partial correlation coefficients. Commun. Stat. Appl. Methods.

[39] Breiman L (2001). Random forests. Mach. Learn..

[40] Debeer D, Strobl C (2020). Conditional permutation importance revisited. BMC Bioinform..

[41] Strobl C, Boulesteix A-L, Kneib T, Augustin T, Zeileis A (2008). Conditional variable importance for random forests. BMC Bioinform..

[42] van der Laan MJ (2006). Statistical inference for variable importance. Int. J. Biostat..

[43] Haensler A, Saeed F, Jacob D (2013). Assessing the robustness of projected precipitation changes over central Africa on the basis of a multitude of global and regional climate projections. Clim. Change.

[44] Oyerinde GT, Lawin AE, Adeyeri OE (2021). Multi-variate infilling of missing daily discharge data on the Niger basin. Water Pract. Technol..

[45] Sen PK (1968). Estimates of the regression coefficient based on Kendall's tau. J. Am. Stat. Assoc..

[46] Adeyeri OE, Ishola KA (2021). Variability and trends of actual evapotranspiration over West Africa: The role of environmental drivers. Agric. For. Meteorol..

[47] Ehret U, Zehe E, Wulfmeyer V, Warrach-Sagi K, Liebert J (2012). HESS opinions "Should we apply bias correction to global and regional climate model data?". Hydrol. Earth Syst. Sci..

[48] Eden JM, Widmann M, Grawe D, Rast S (2012). Skill, correction, and downscaling of GCM-simulated precipitation. J. Clim..

[49] Maraun D (2012). Nonstationarities of regional climate model biases in European seasonal mean temperature and precipitation sums. Geophys. Res. Lett..

[50] Lewis SC, Karoly DJ (2013). Evaluation of historical diurnal temperature range trends in CMIP5 models. J. Clim..

[51] Wang K, Clow GD (2020). The diurnal temperature range in CMIP6 models: climatology, variability, and evolution. J. Clim..

[52] Lindvall J, Svensson G (2015). The diurnal temperature range in the CMIP5 models. Clim. Dyn..

[53] Zhou L (2004). Evidence for a significant urbanization effect on climate in China. Proc. Natl. Acad. Sci. U.S.A..

[54] Wild M (2005). From dimming to brightening: decadal changes in solar radiation at Earth's surface. Science.

[55] Grose MR, Foster S, Risbey JS, Osbrough S, Wilson L (2019). Using indices of atmospheric circulation to refine southern Australian winter rainfall climate projections. Clim. Dyn..

[56] Srivastava A, Grotjahn R, Ullrich PA (2020). Evaluation of historical CMIP6 model simulations of extreme precipitation over contiguous US regions. Wea. Clim. Extrem..

[57] Alexander LV, Arblaster JM (2017). Historical and projected trends in temperature and precipitation extremes in Australia in observations and CMIP5. Wea. Clim. Extrem..

[58] Sera F (2019). How urban characteristics affect vulnerability to heat and cold: A multi-country analysis. Int. J. Epidemiol..

[59] Adeyeri OE, Akinsanola AA, Ishola KA (2017). Investigating surface urban heat island characteristics over Abuja, Nigeria: Relationship between land surface temperature and multiple vegetation indices. Remote Sens. Appl. Soc. Environ..

[60] Ishola KA, Okogbue EC, Adeyeri OE (2016). A quantitative assessment of surface urban heat islands using satellite multitemporal data over Abeokuta, Nigeria. Int. J. Atmos. Sci..

[61] Li D, Bou-Zeid E (2013). Synergistic interactions between urban heat islands and heat waves: The impact in cities is larger than the sum of its parts. J. Appl. Meteorol. Climatol..

[62] Clemens KK (2021). Evaluating the association between extreme heat and mortality in urban Southwestern Ontario using different temperature data sources. Sci. Rep..

[63] Adeyeri OE, Laux P, Lawin AE, Arnault J (2020). Assessing the impact of human activities and rainfall variability on the river discharge of Komadugu-Yobe Basin, Lake Chad Area. Environ. Earth Sci..

[64] Miralles DG, Gentine P, Seneviratne SI, Teuling AJ (2019). Land-atmospheric feedbacks during droughts and heatwaves: State of the science and current challenges. Ann. N. Y. Acad. Sci..

[65] Adeyeri OE, Laux P, Lawin AE, Ige SO, Kunstmann H (2020). Analysis of hydrometeorological variables over the transboundary Komadugu-Yobe basin, West Africa. J. Water Clim. Change.

[66] Berg P (2009). Seasonal characteristics of the relationship between daily precipitation intensity and surface temperature. J. Geophys. Res..

[67] Adeyeri OE (2022). Homogenising meteorological variables: Impact on trends and associated climate indices. J. Hydrol..

[68] Tegegne G, Melesse AM, Alamirew T (2021). Projected changes in extreme precipitation indices from CORDEX simulations over Ethiopia, East Africa. Atmos. Res..

[69] R Core Team. R: A language and environment for statistical computing. *R Foundation for Statistical Computing*, Vienna, Austria, version **4.1.2** (2021). Available at https://www.R-project.org/.

[70] Python Software Foundation. *Python Language Reference*, version **3.9** (2022). Available at https://www.python.org/.

