# Publisher Correction: The trend and spatial spread of multisectoral climate extremes in CMIP6 models

**DOI:** 10.1038/s41598-023-28115-z

**Published:** 2023-01-19

**Authors:** Oluwafemi E. Adeyeri, Wen Zhou, Xuan Wang, Ruhua Zhang, Patrick Laux, Kazeem A. Ishola, Muhammad Usman

**Affiliations:** 1 School of Energy and Environment, City University of Hong Kong, Kowloon, Hong Kong SAR China; 2 Department of Atmospheric and Oceanic Sciences & Institute of Atmospheric Sciences, Fudan University, Shanghai, China; 3 Center for Ocean Research in Hong Kong and Macau (CORE), Hong Kong, China; 4 Institute for Meteorology and Climate Research Atmospheric Environmental Research, Karlsruhe Institute of Technology, Campus Alpine, Germany; 5 Irish Climate Analysis and Research UnitS (ICARUS), Department of Geography, Maynooth University, Maynooth, Ireland; 6 School of Engineering, Faculty of Science Engineering and Built Environment, Deakin University, Geelong, Australia

Correction to: *Scientific Reports* 10.1038/s41598-022-25265-4, published online 05 December 2022

In the original version of this Article Oluwafemi E. Adeyeri was incorrectly affiliated with ‘Department of Atmospheric and Oceanic Sciences & Institute of Atmospheric Sciences, Fudan University, Shanghai, China’. Additionally, Wen Zhou was incorrectly affiliated with ‘Center for Ocean Research in Hong Kong and Macau (CORE), Hong Kong, China’. The correct affiliations are listed below.

**Oluwafemi E. Adeyeri:**

School of Energy and Environment, City University of Hong Kong, Kowloon, Hong Kong, SAR, China.

Center for Ocean Research in Hong Kong and Macau (CORE), Hong Kong, SAR, China.

**Wen Zhou:**

Department of Atmospheric and Oceanic Sciences & Institute of Atmospheric Sciences, Fudan University, Shanghai, China.

The original Article has been corrected.

