# Supplementary material for: The trend and spatial spread of multisectoral climate extremes in CMIP6 models
Source: Sci Rep. 2022 Dec 5;12:21000. doi: 10.1038/s41598-022-25265-4 (PMC9722700; doi:10.1038/s41598-022-25265-4)
Supplement: Supplementary file 1 — Supplementary Information. [file 41598_2022_25265_MOESM1_ESM.docx]

**The** **trend and spatial spread of multisectoral climate extremes in CMIP6 models**

Oluwafemi E. Adeyeri ^a,c^, Wen Zhou ^b*^, Xuan Wang ^a^, Zhang H. Ruhua^b^, Patrick Laux. ^d^, Kazeem A. Ishola ^e^, Muhammad Usman ^f^

^a^ School of Energy and Environment, City University of Hong Kong, Kowloon, Hong Kong Special Administrative Region

^b^ Department of Atmospheric and Oceanic Sciences & Institute of Atmospheric Sciences, Fudan University, Shanghai, China

^c^ Center for Ocean Research in Hong Kong and Macau (CORE)

^d^ Institute for Meteorology and Climate Research Atmospheric Environmental Research, Karlsruhe Institute of Technology, Campus Alpine, Germany

^e^ Irish Climate Analysis and Research UnitS (ICARUS), Department of Geography, Maynooth University, Maynooth, Ireland

^f^ School of Engineering, Faculty of Science Engineering and Built Environment, Deakin University, Geelong, Australia.

*^*^Corresponding author: Wen Zhou, wen_zhou@fudan.edu.cn. Department of Atmospheric and Oceanic Sciences and Institute of Atmospheric Sciences, Fudan University, Shanghai, China*


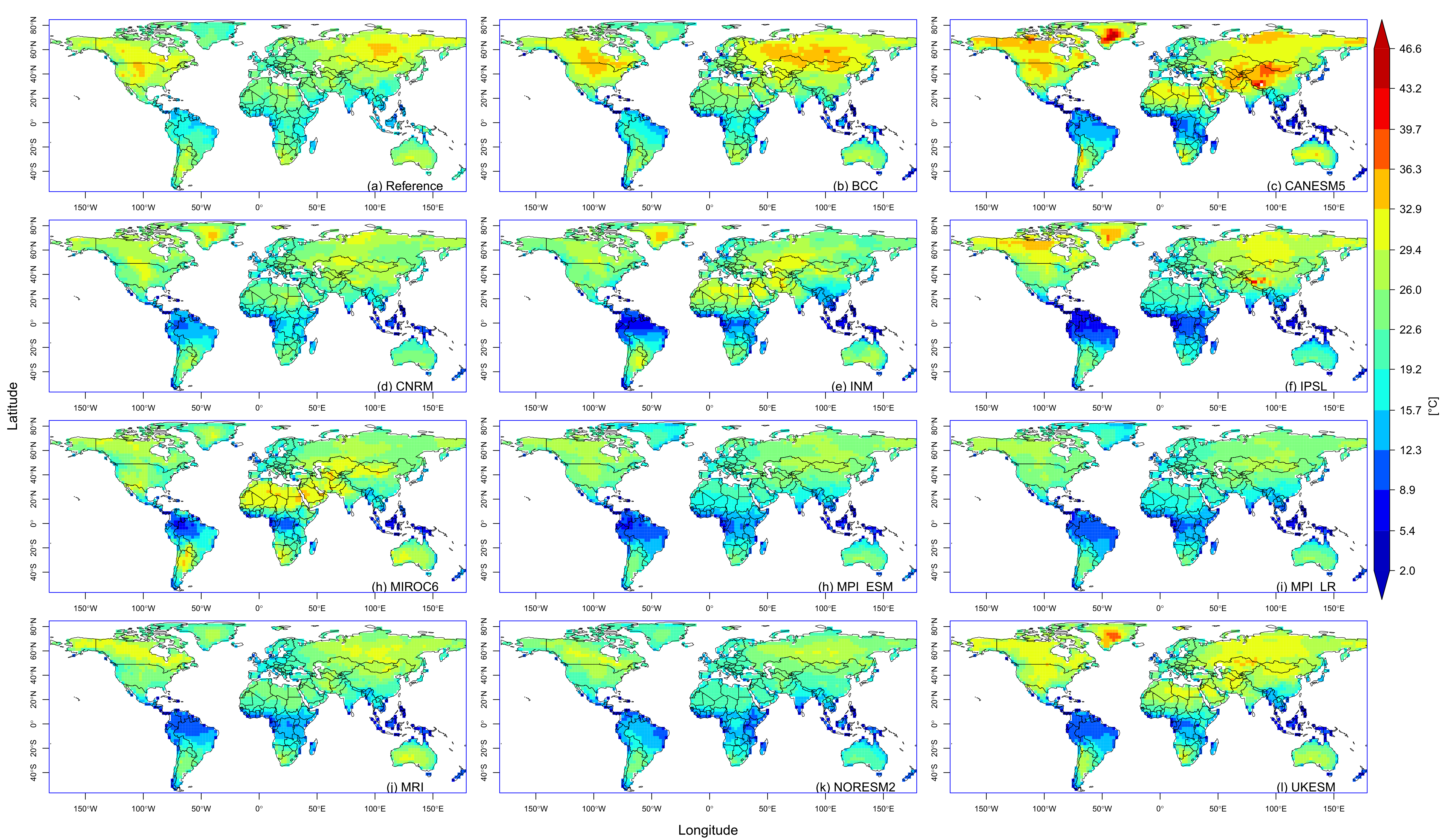


Figure S1: Climatology of ETR for different CMIP6 models. Figure was drawn in the R version 4.1.2 Platform [69] (https://www.R-project.org)


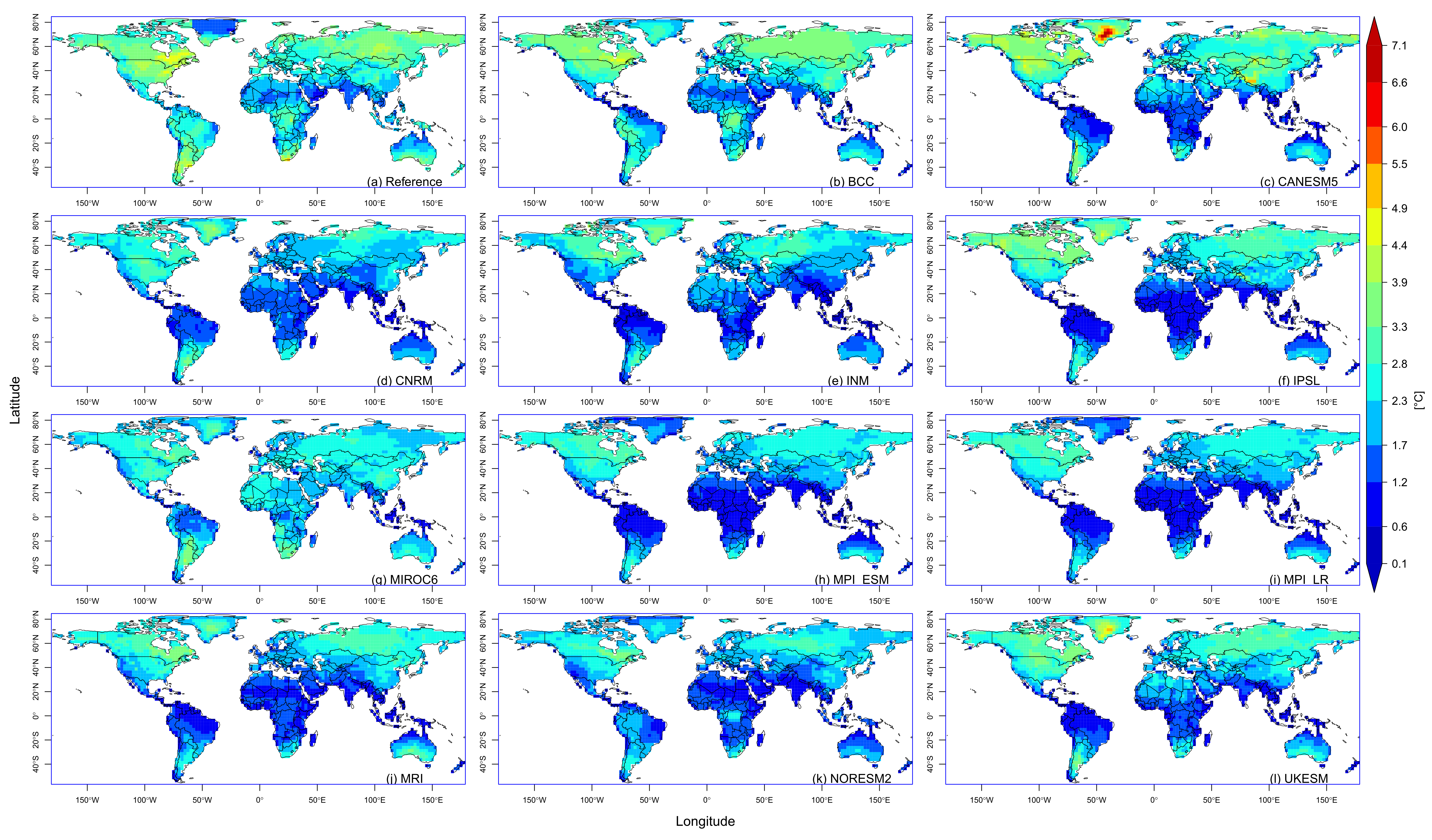


Figure S2: Climatology of DTRV for different CMIP6 models. Figure was drawn in the R version 4.1.2 Platform [69] (https://www.R-project.org)


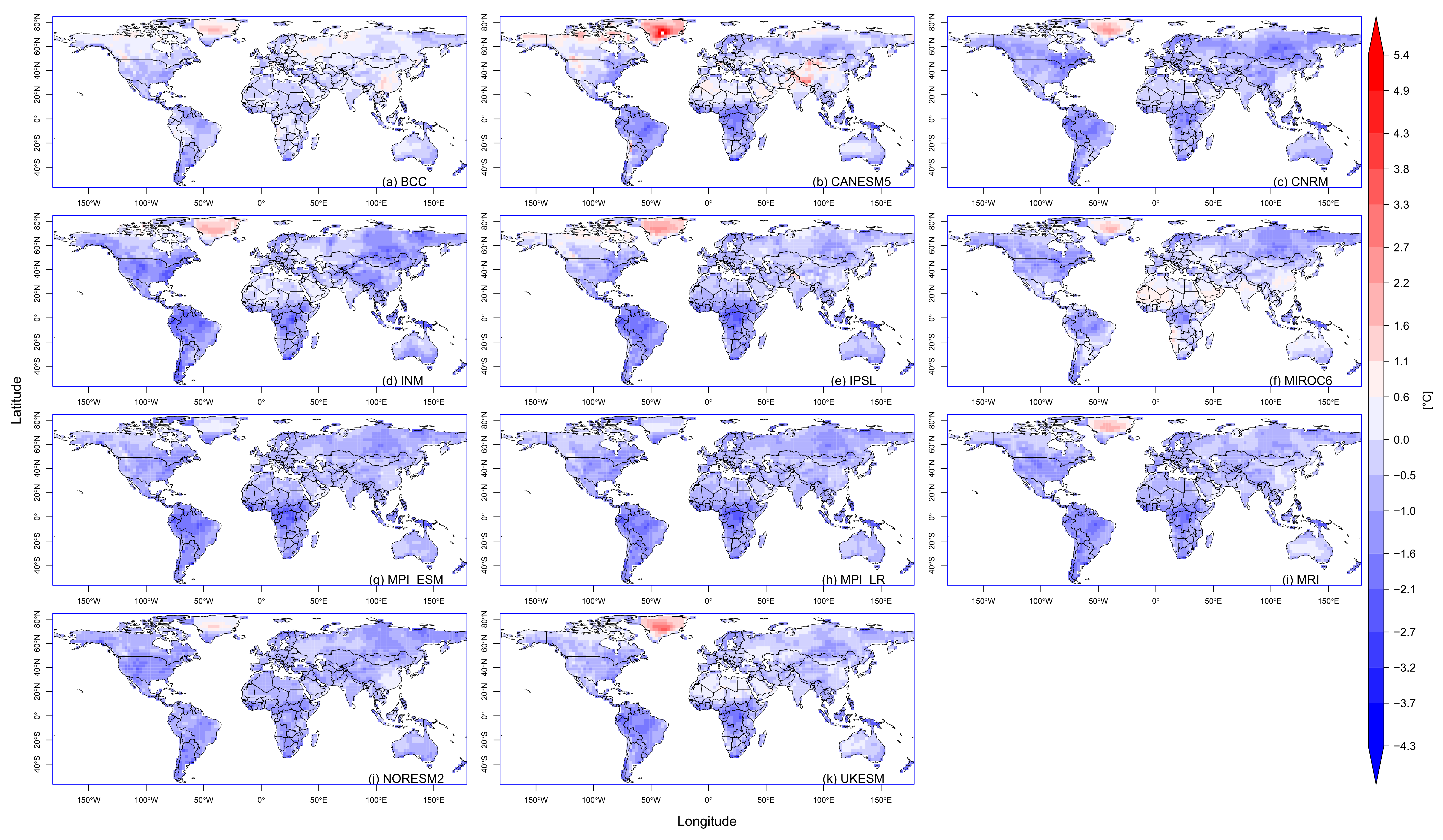


Figure S3: Bias of DTRV climatology for different CMIP6 models. Figure was drawn in the R version 4.1.2 Platform [69] (https://www.R-project.org)


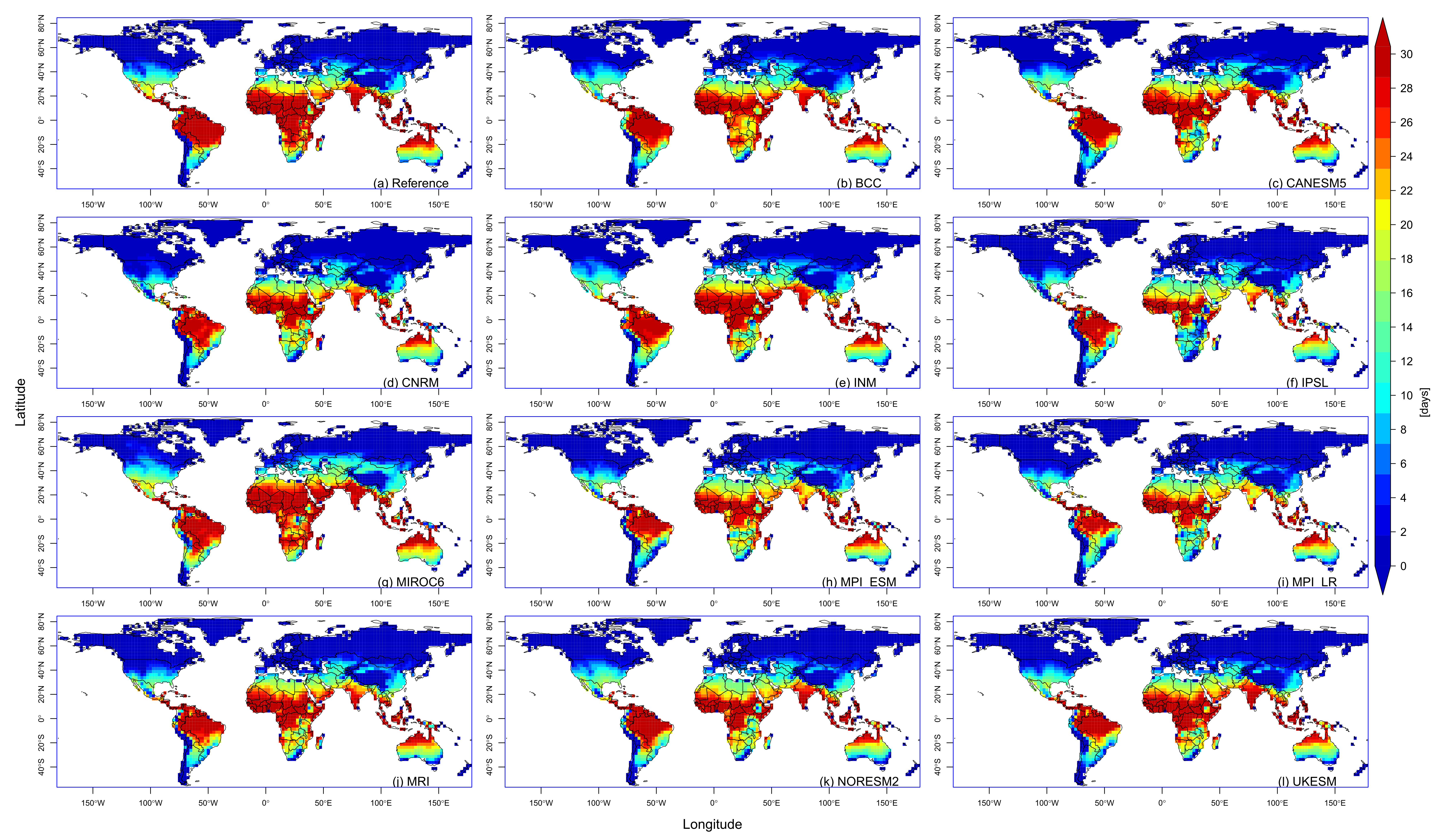


Figure S4: Climatologyof HWI for different CMIP6 models. Figure was drawn in the R version 4.1.2 Platform [69] (https://www.R-project.org)


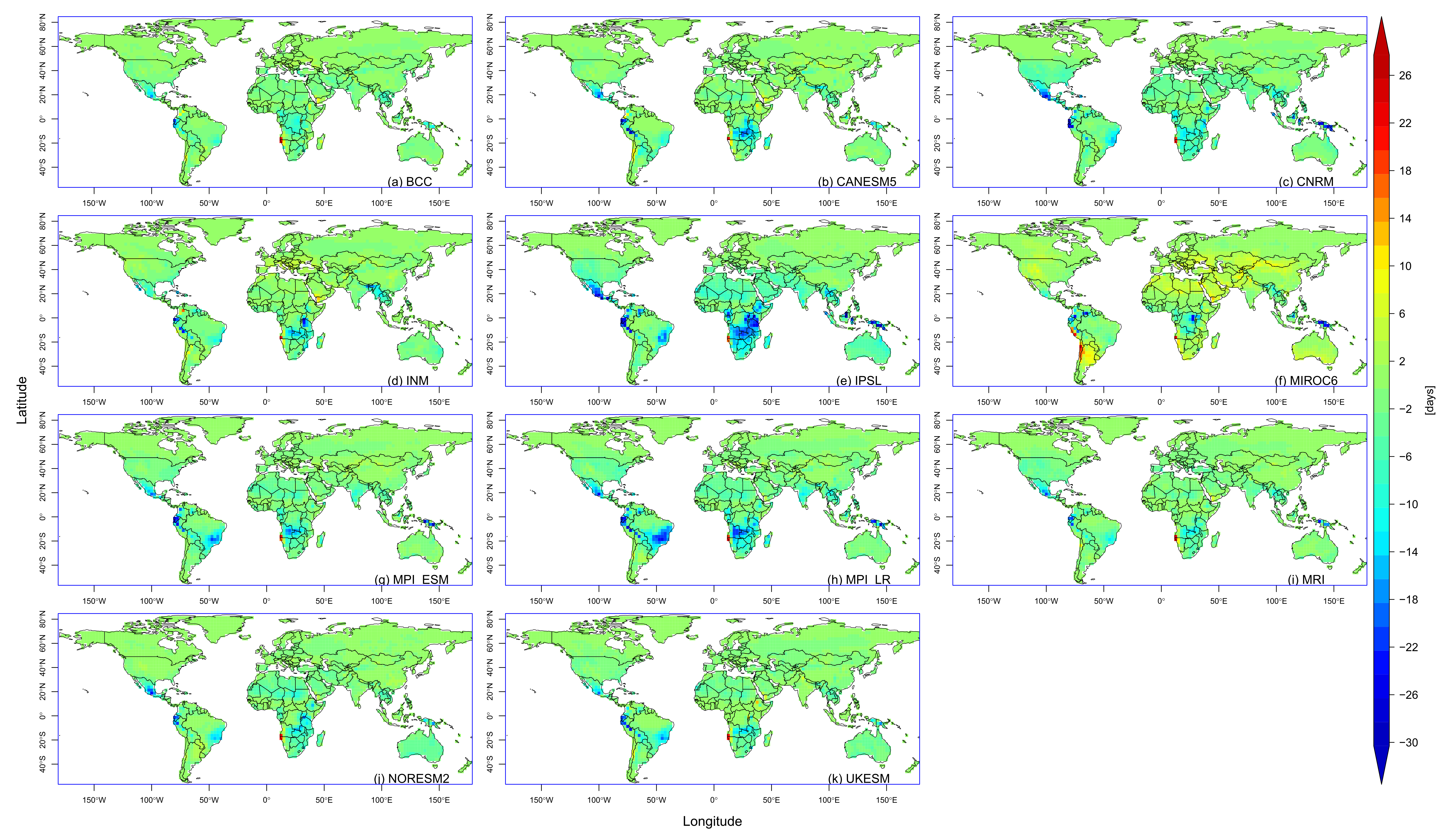


Figure S5: Bias of HWI climatology for different CMIP6 models. Figure was drawn in the R version 4.1.2 Platform [69] (https://www.R-project.org)


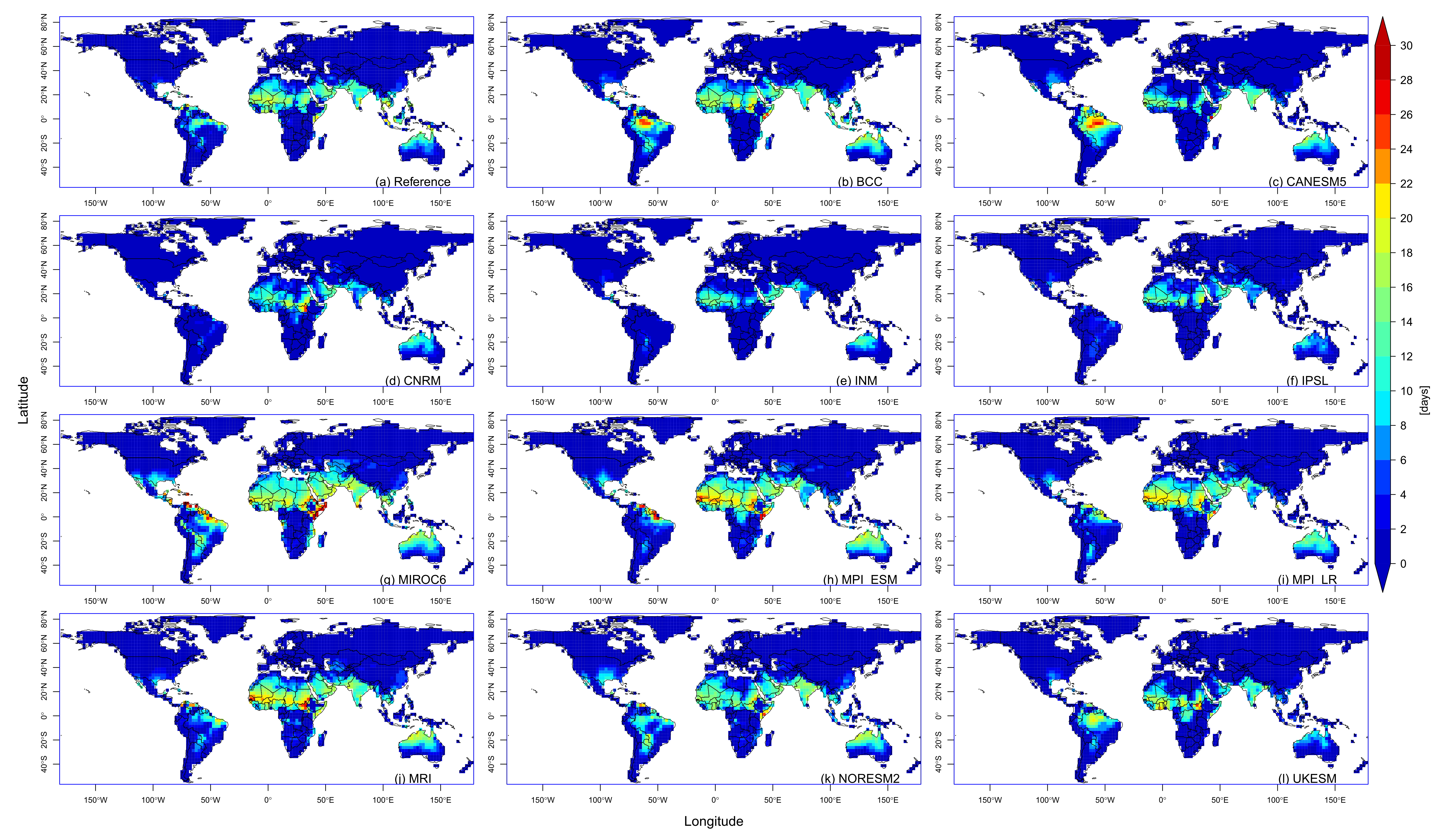


Figure S6: Climatology of DSTL for different CMIP6 models. Figure was drawn in the R version 4.1.2 Platform [69] (https://www.R-project.org)


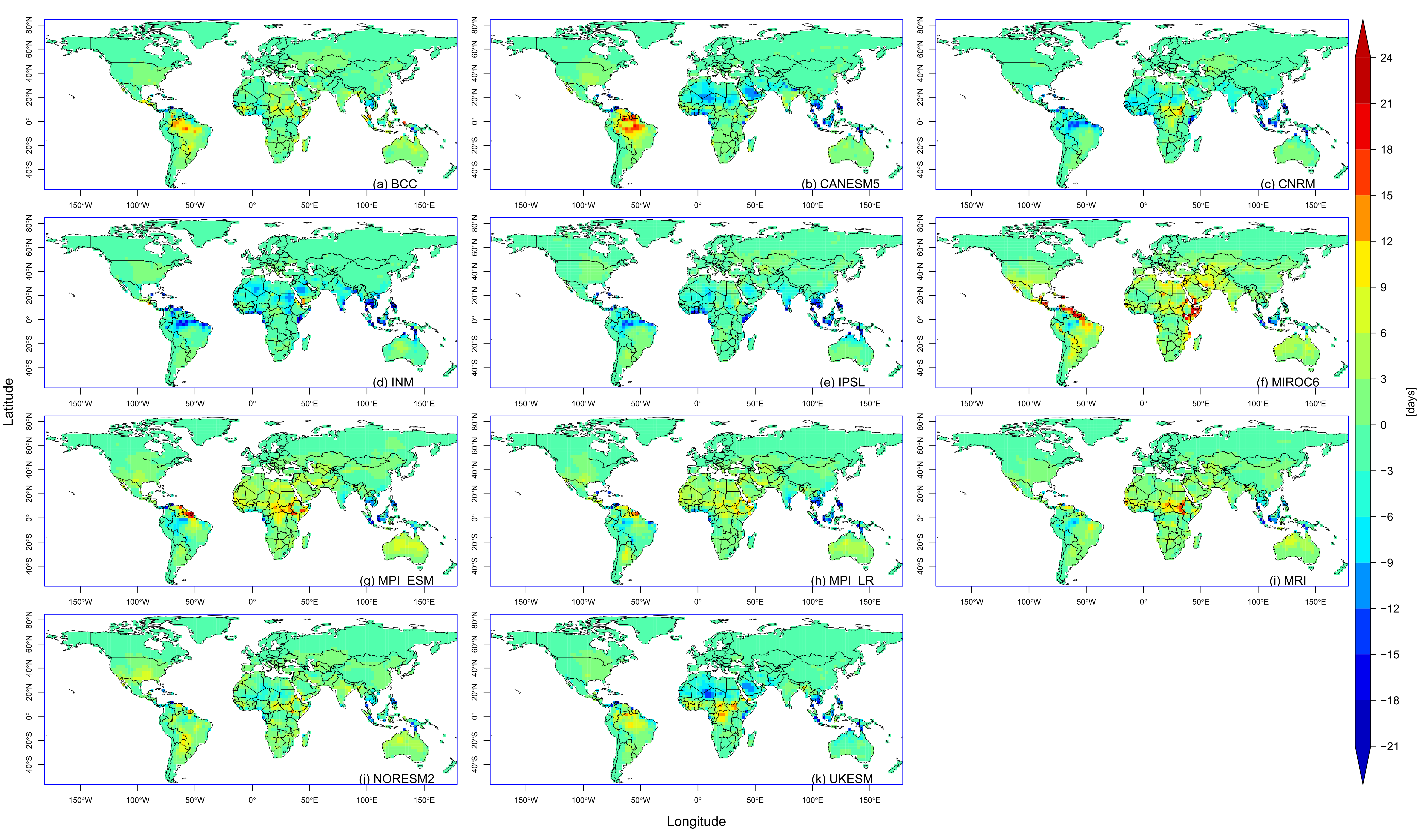


Figure S7: Bias of DSTL climatology for different CMIP6 models. Figure was drawn in the R version 4.1.2 Platform [69] (https://www.R-project.org)


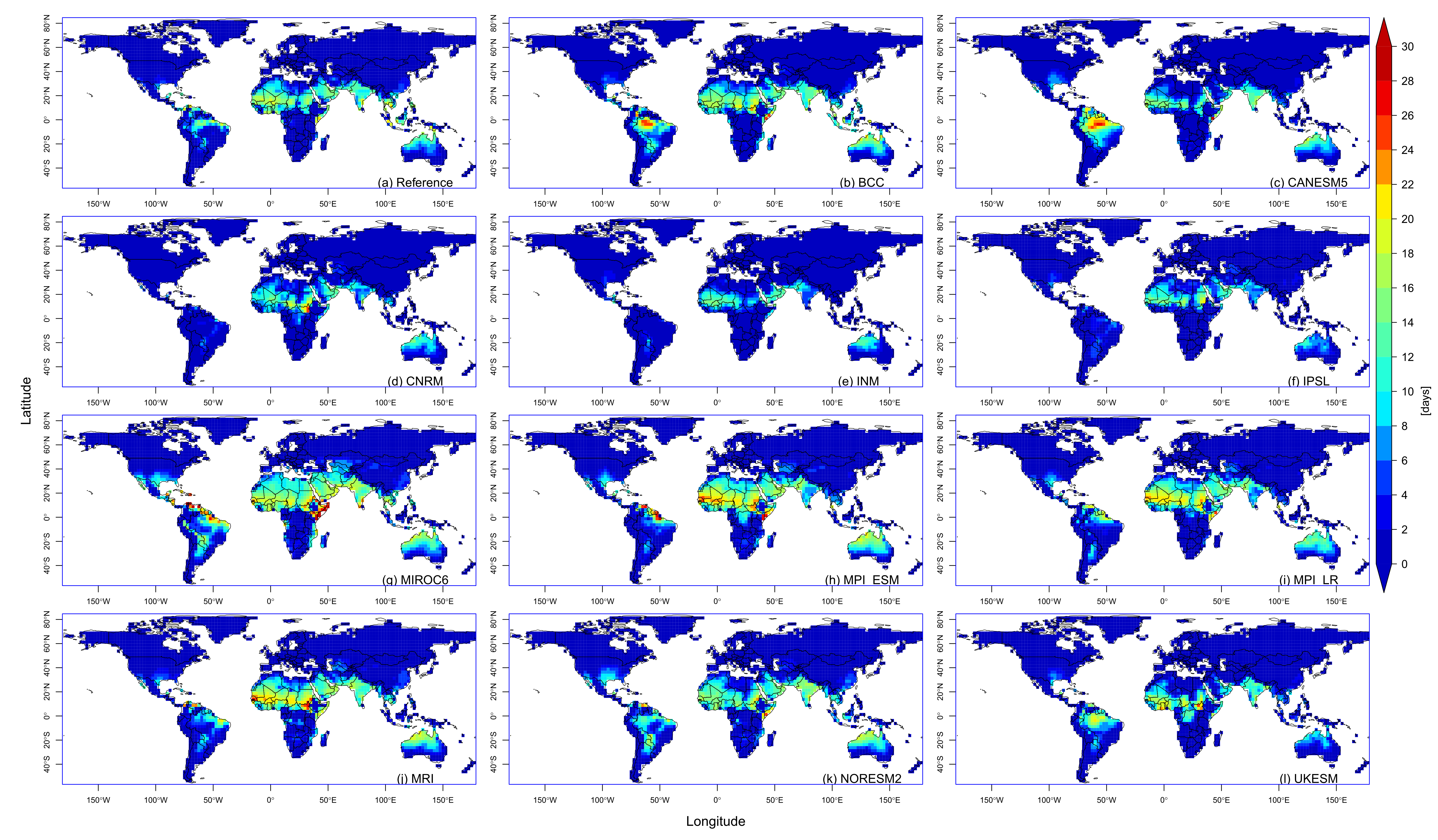


Figure S8: Climatology of HWTL for different CMIP6 models. Figure was drawn in the R version 4.1.2 Platform [69] (https://www.R-project.org)


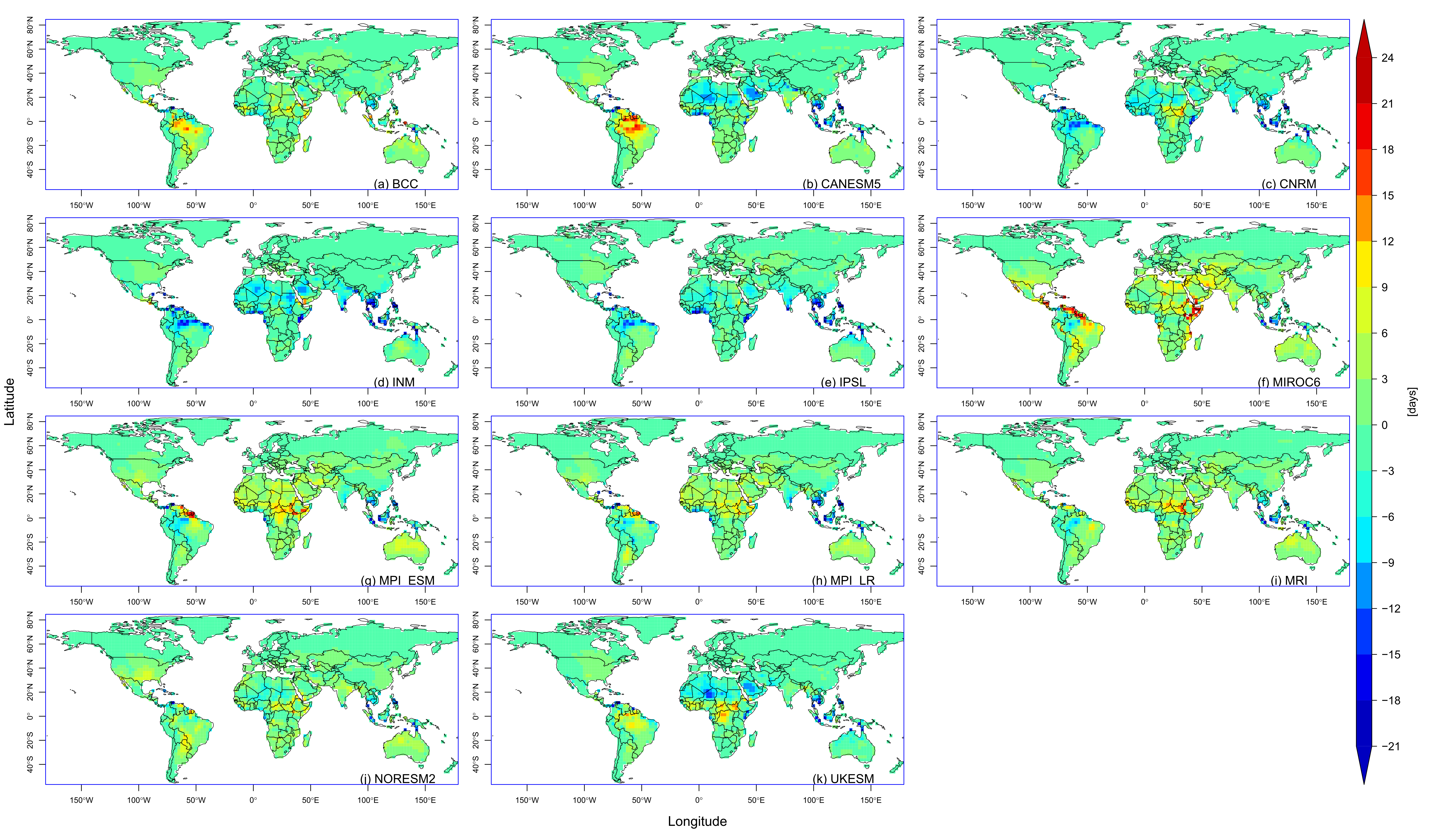


Figure S9: Bias of HWTL climatology for different CMIP6 models. Figure was drawn in the R version 4.1.2 Platform [69] (https://www.R-project.org)


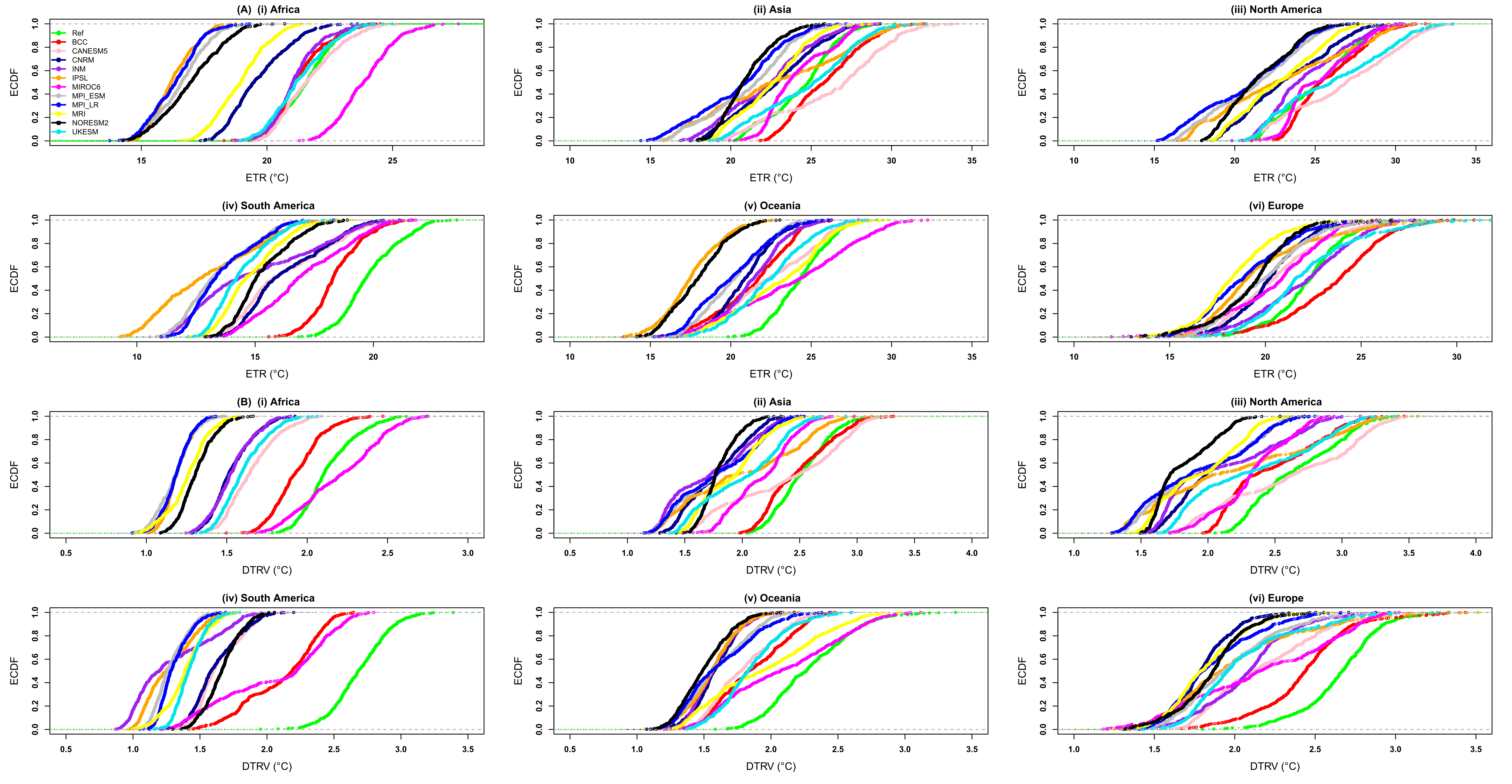


Figure S10: ECDF for (a) ETR, (b) DTRV


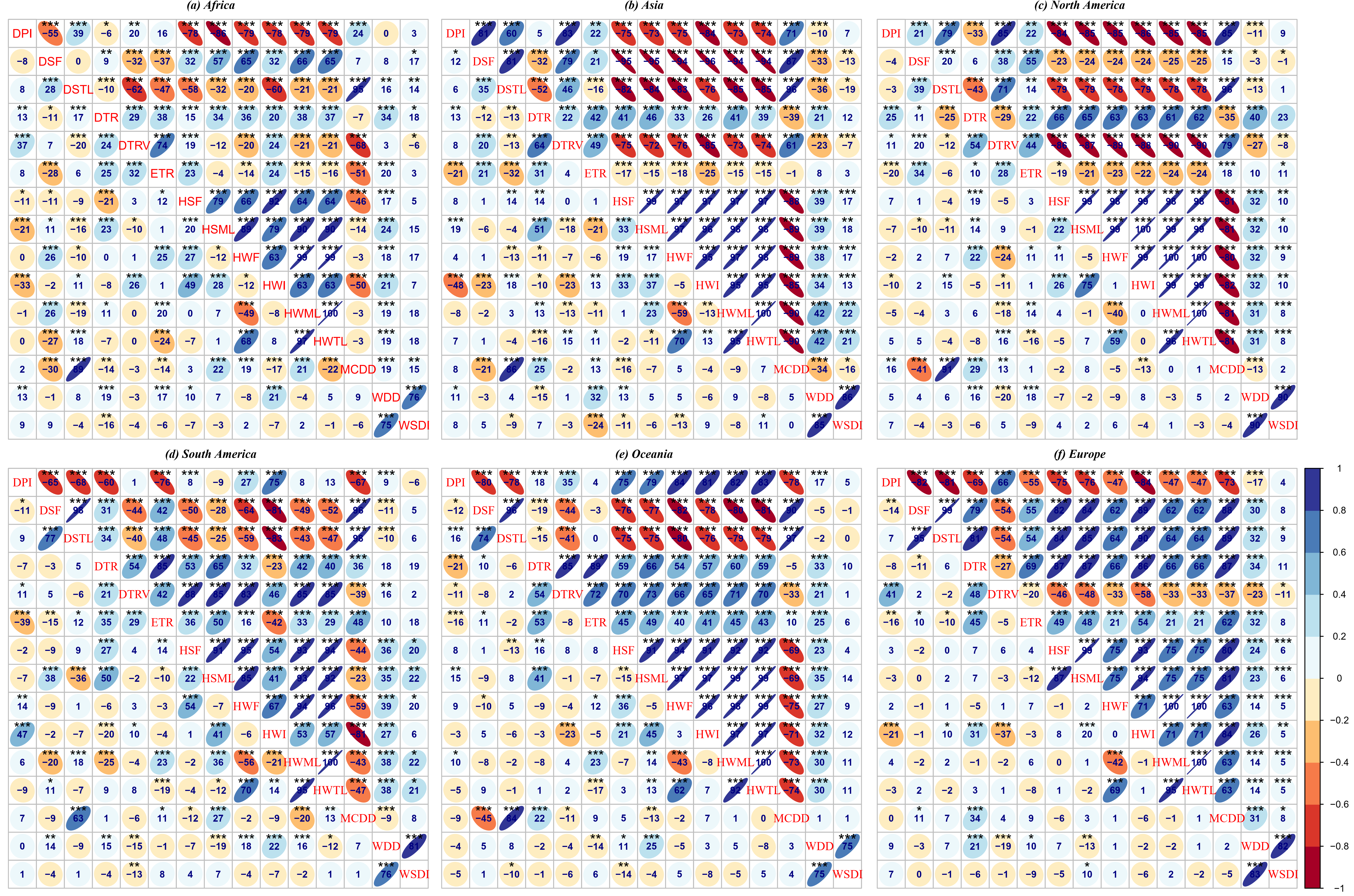


Figure S11: As in S11 but for the model ensemble mean during the historical period.


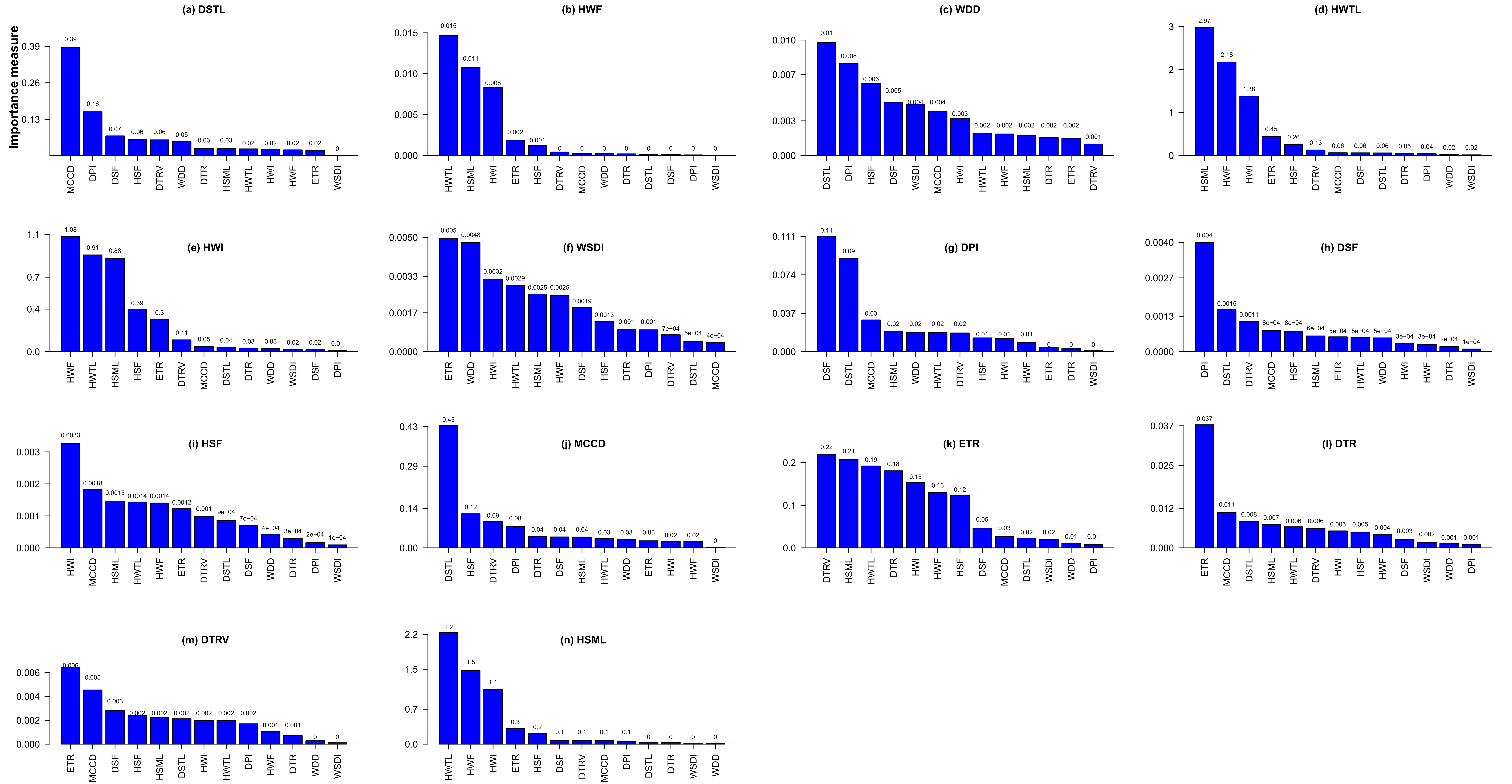


Figure S12: Conditional index of importance of extreme indices over Africa using the reference


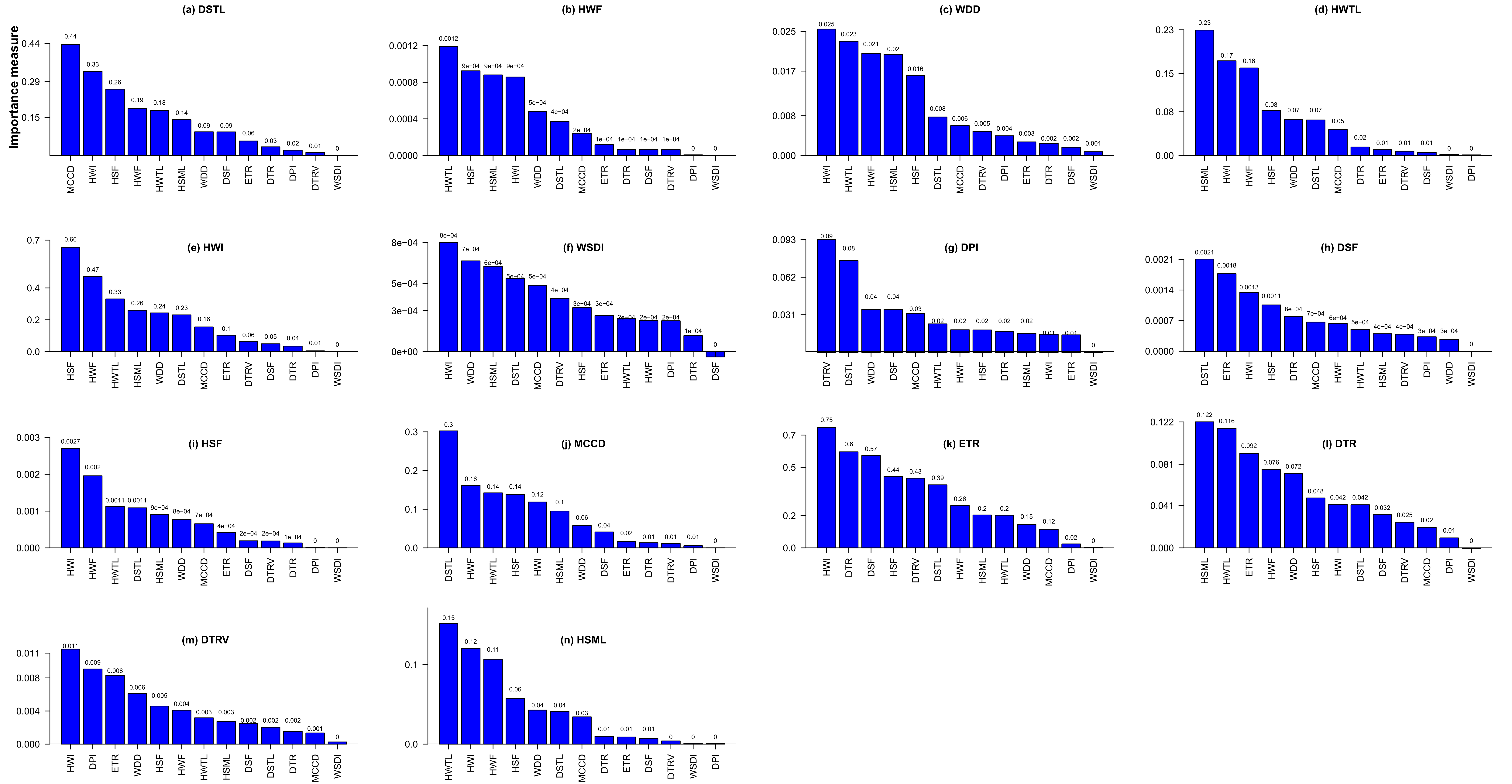


Figure S13: Conditional index of importance of extreme indices over Asia using the reference


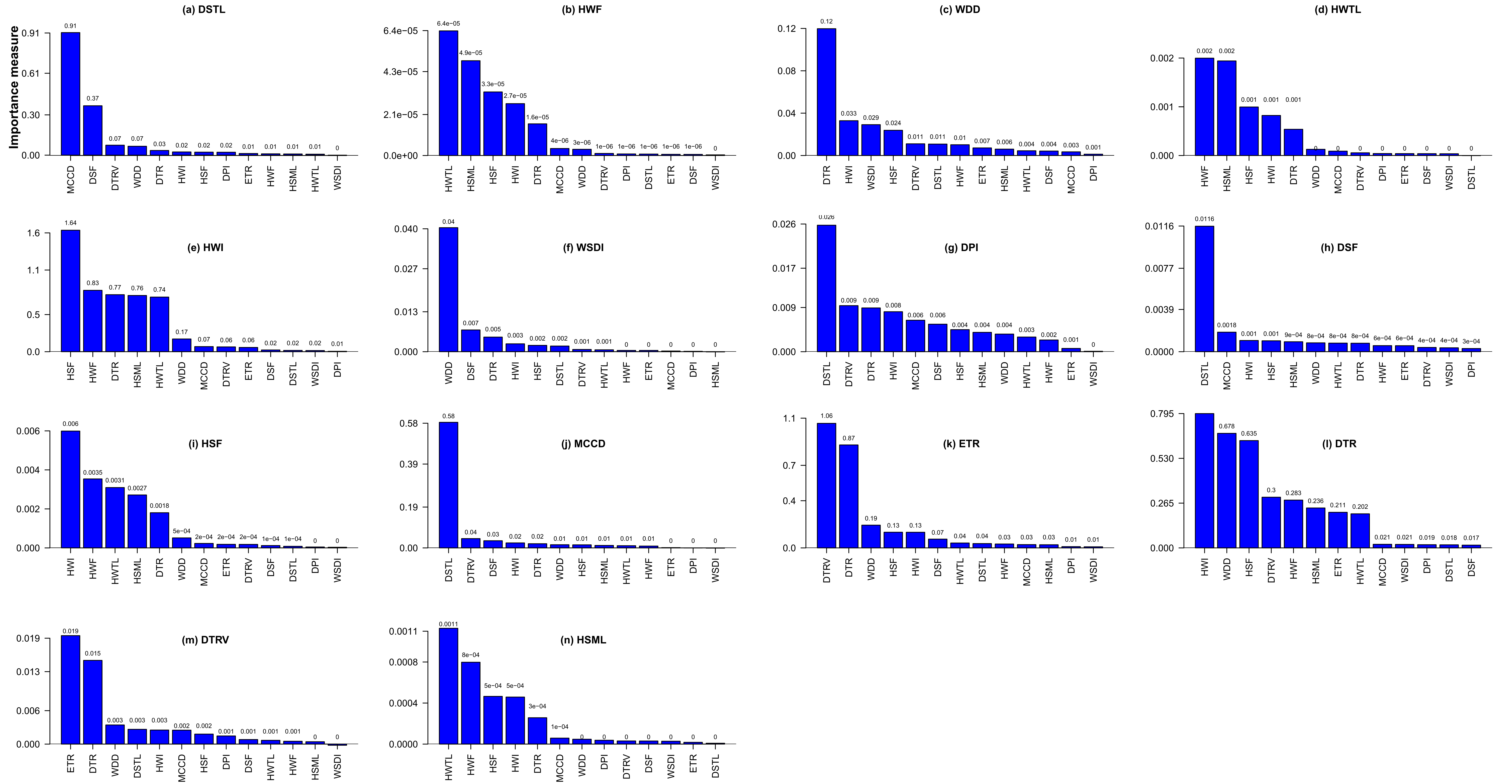


Figure S14: Conditional index of importance of extreme indices over Europe using the reference


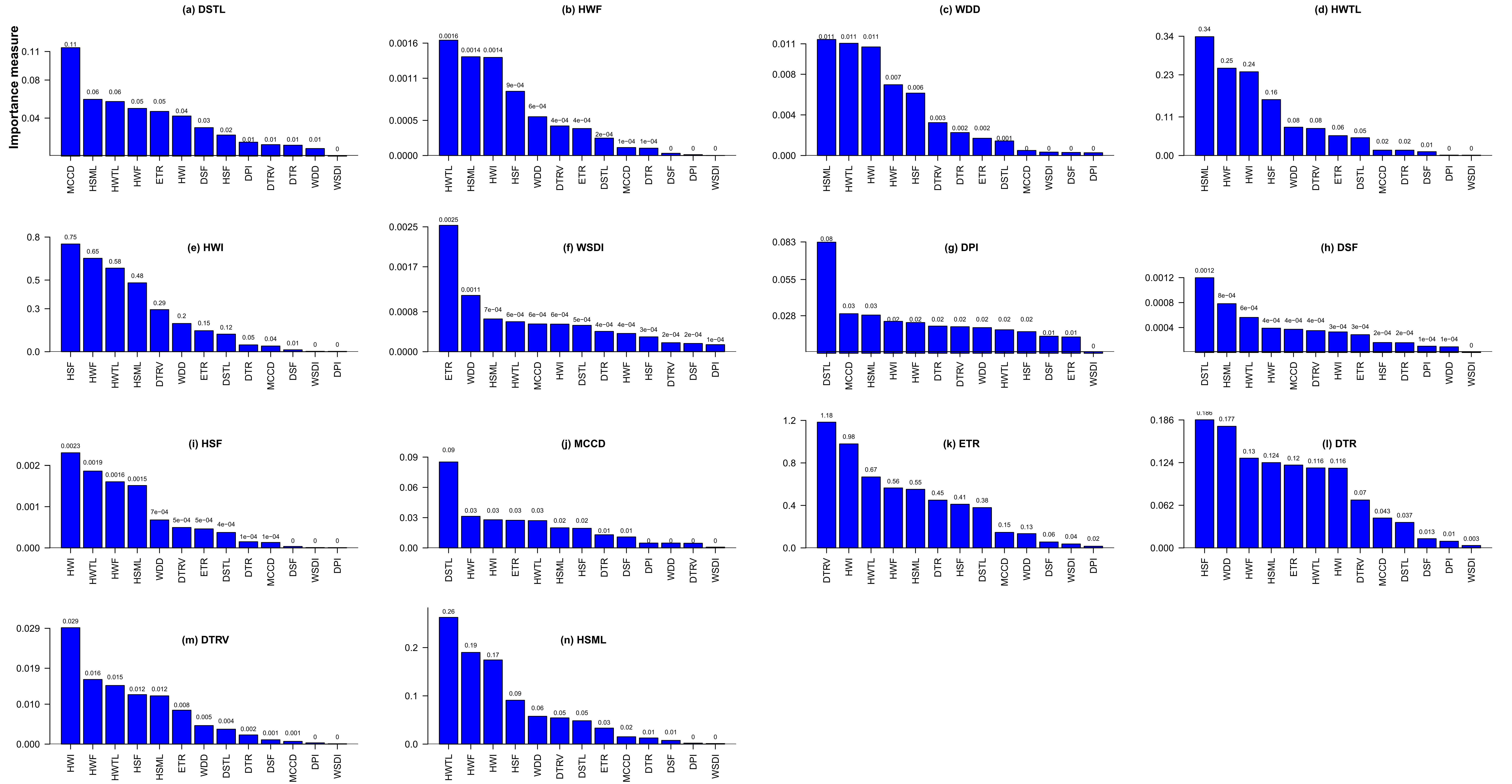


Figure S15: Conditional index of importance of extreme indices over North America using the reference


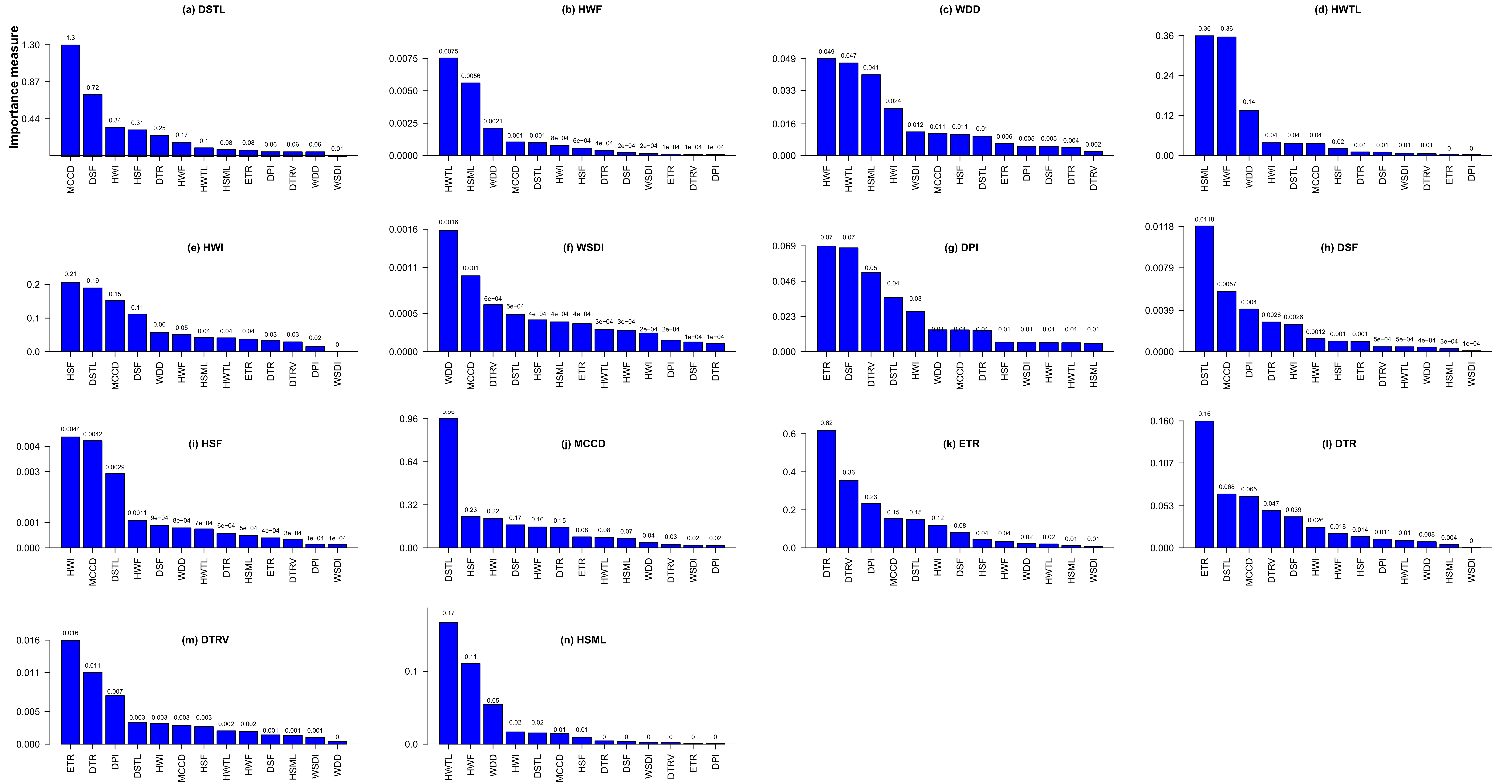


Figure S16: Conditional index of importance of extreme indices over South America using the reference


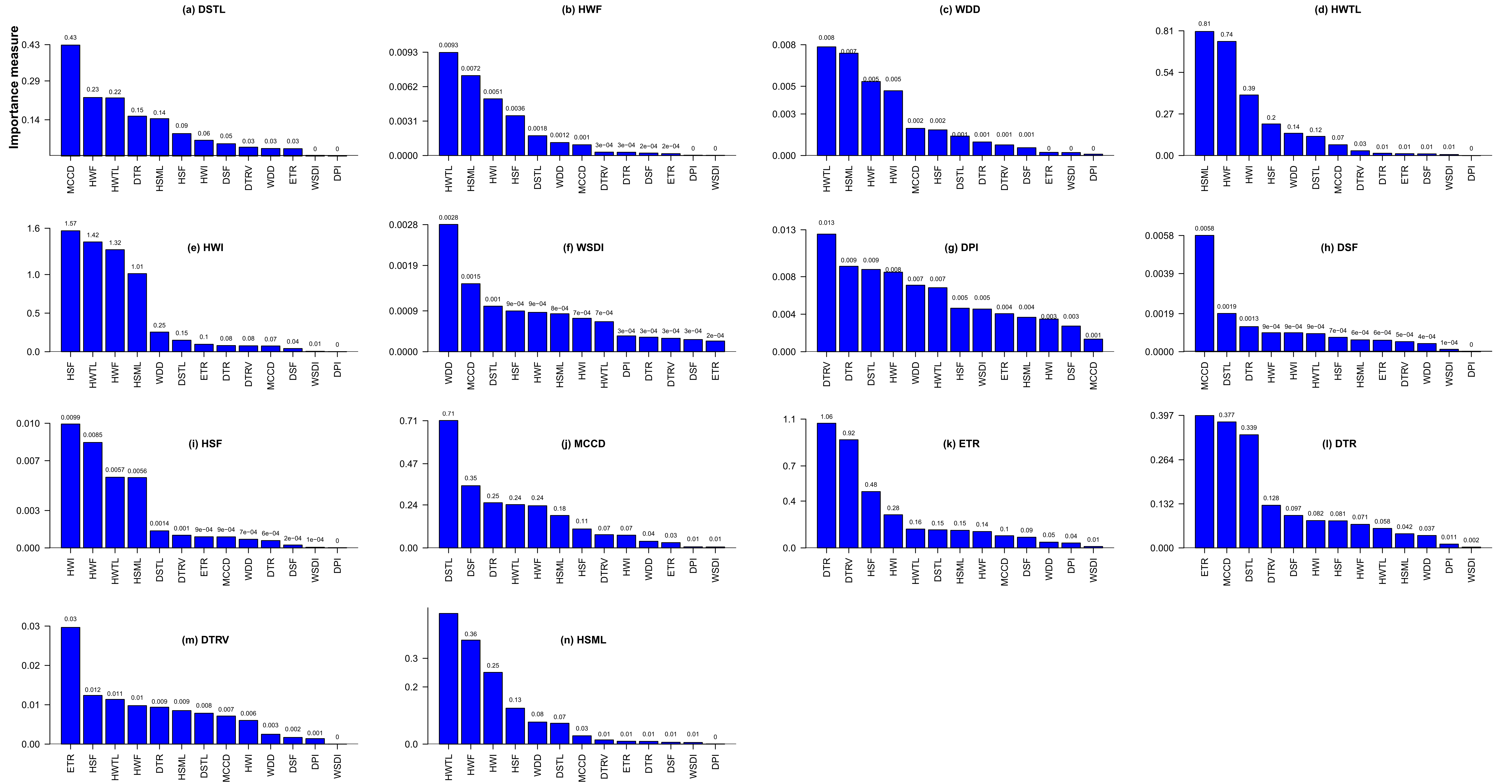


Figure S17: Conditional index of importance of extreme indices over Oceania using the reference


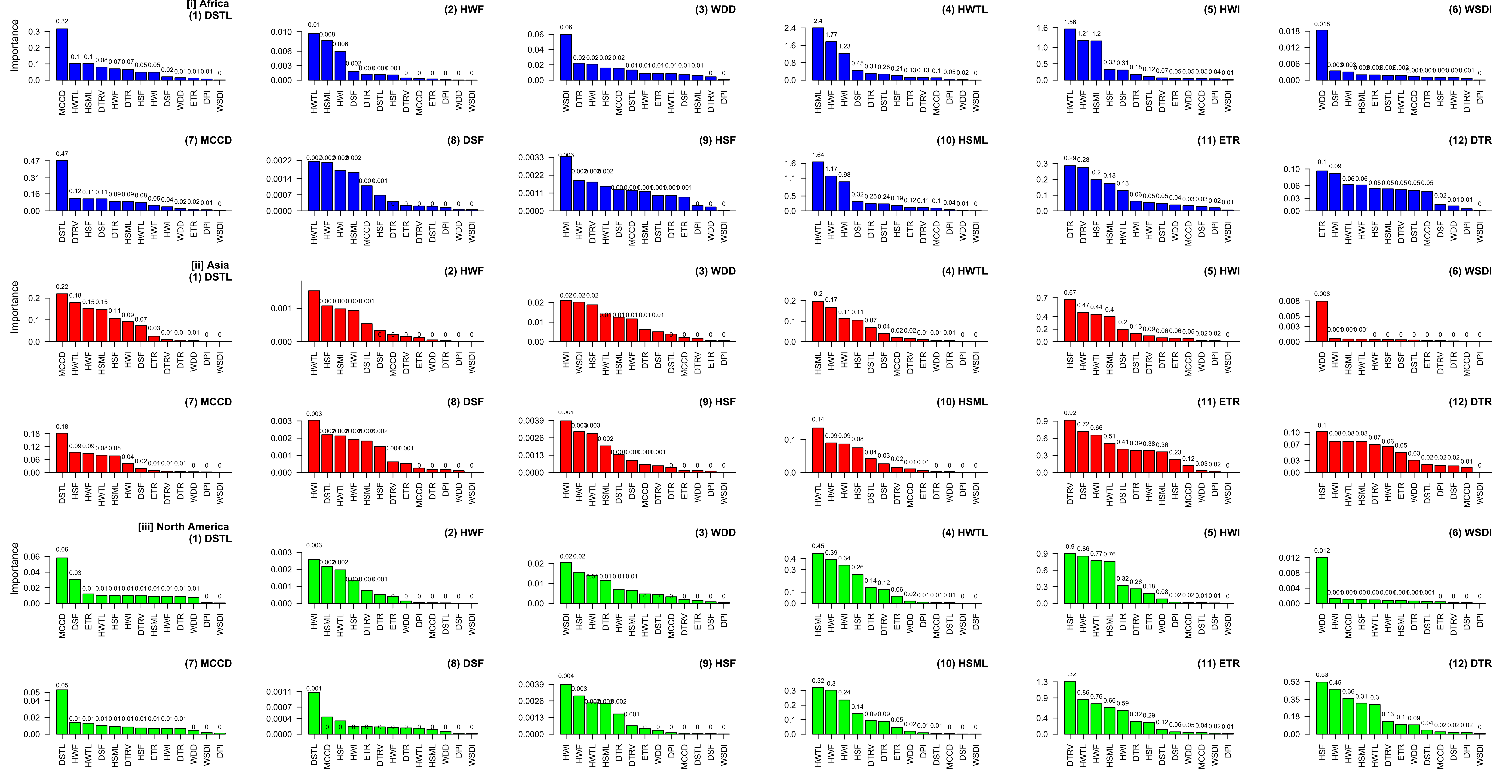


Figure S18: Conditional index of importance of extreme indices using the historical CMIP6 ensemble mean


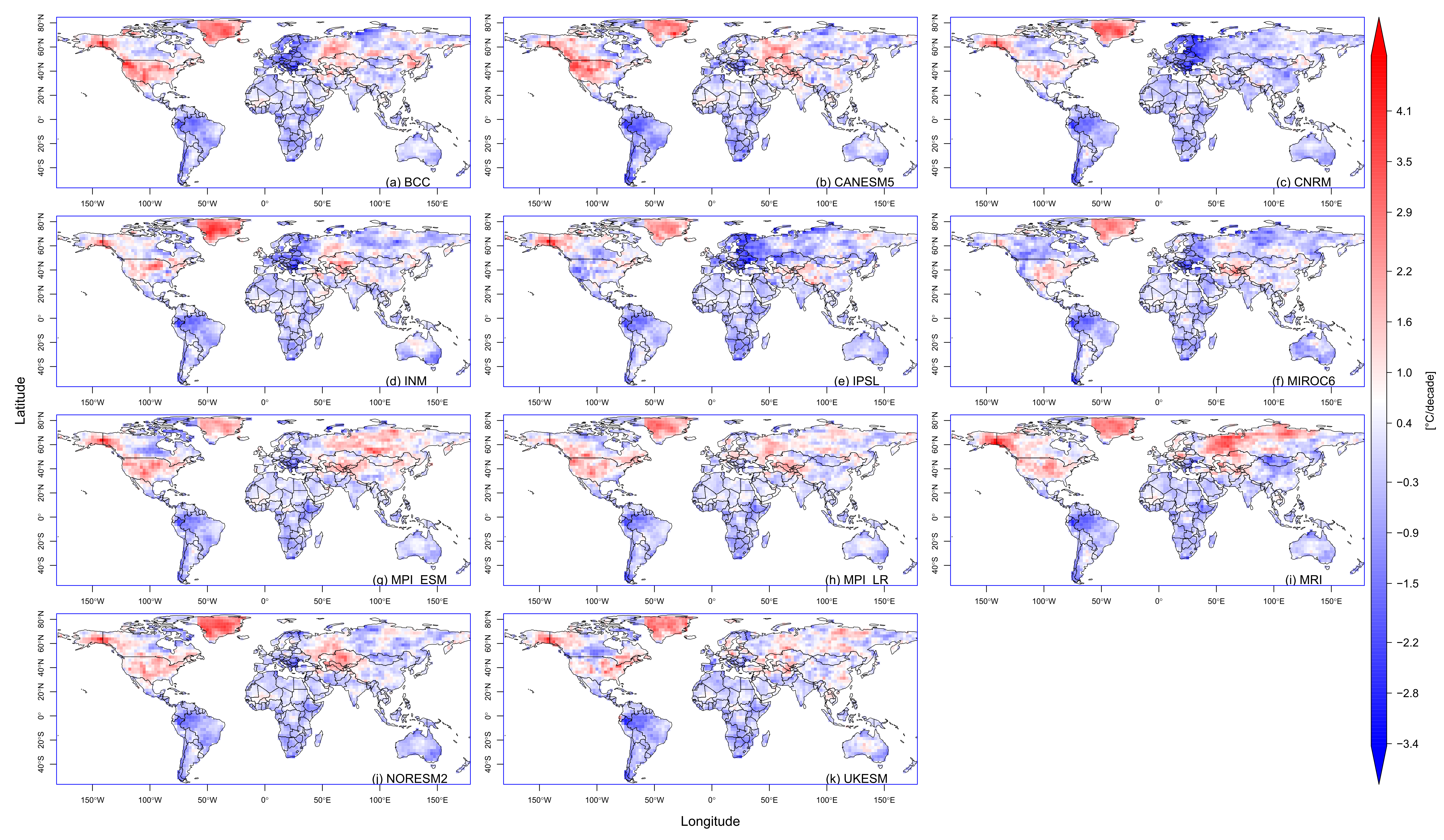


Figure S19: ETR trend bias for CMIP6 models during the historical period. Figure was drawn in the R version 4.1.2 Platform [69] (https://www.R-project.org)


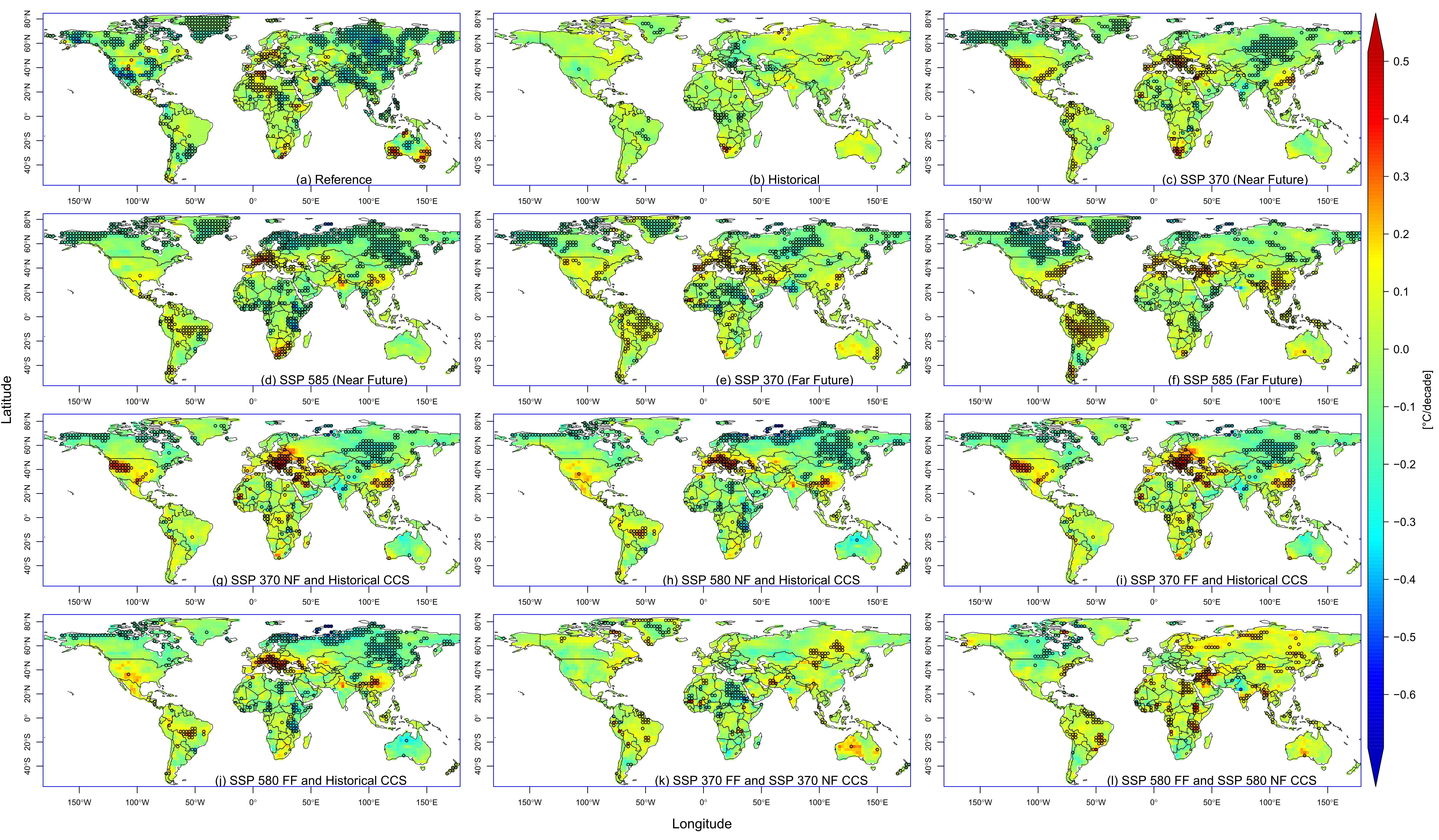


Figure S20: Trends of DTR during the historical and future periods (a-f), climate change signals in trends between the historical, near future and far future (g-l) for both SSP 370 and 585. Hatchings represent area with significant trend at 95% confidence level. Figure was drawn in the R version 4.1.2 Platform [69] (https://www.R-project.org)

Table S1: List of CMIP6 coupled atmosphere-ocean climate models

| S/N | Model | Acronym | Resolution (LonxLat) | Institute (Country) |
| --- | --- | --- | --- | --- |
| 1 | BCC-CSM2-MR | BCC | 320x160 | The Beijing Climate Center Climate System Model, Beijing Climate Center, China Meteorological Administration (China) |
| 2 | CANESM5 | CANESM5 | 128x64 | Canadian Earth System Model version 5, Canadian Centre for Climate Modelling and Analysis (Canada) |
| 3 | CNRM-CM6-1 | CNRM5 | 256x128 | Centre National de Recherches Meteorologiques/Centre Europeen de Recherche et Formation Avancees en Calcul Scientifique (France) |
| 4 | INM-CM5-0 | INM | 180x120 | Institute for Numerical Mathematics, Russian Academy of Science (Russia) |
| 5 | IPSL-CM6A-LR | IPSL | 144x143 | Institut Pierre-Simon Laplace (France) |
| 6 | MIROC6 | MIROC6 | 256x128 | Atmosphere and Ocean Research Institute (The University of Tokyo), National Institute for Environmental Studies, and Japan Agency for Marine-Earth Science and Technology (Japan) |
| 7 | MPI-ESM-1-2HR | MPI-ESM | 384x192 | Max Planck Institute for Meteorology (MPI-M) (Germany) |
| 8 | MPI-ESM-1-LR | MPI-LR | 192x96 | Max Planck Institute for Meteorology (MPI-M) (Germany) |
| 9 | MRI-ESM2-0 | MRI | 320x160 | Meteorological Research Institute (Japan) |
| 10 | NORESM2-MM | NORESM2 | 288x192 | Norwegian Climate Centre (Norway) |
| 11 | UKESM1-0-LL | UKESM | 189×138 | UK Met Office and NERC research centres (United Kingdom) |

Table S2: Best-performing models on the continental scale

| Indices | Africa | Asia | Europe | NORTH America | SOUTH America | Oceania | Most suitable |
| --- | --- | --- | --- | --- | --- | --- | --- |
| **DPI** | MPI-ESM | MPI-LR | MPI-LR | MPI-LR | MPI-LR | MPI-LR | MPI-LR (5) |
| **DTR** | UKESM | CANESM5 | INM | CANESM5 | BCC | CANESM5 | CANESM5 (3) |
| **DTRV** | MIROC6 | BCC | BCC | BCC | BCC | BCC | BCC(5) |
| **DSF** | IPSL | IPSL | UKESM | MPI-ESM | INM/IPSL | IPSL | IPSL(4) |
| **DSTL** | IPSL | CANESM5 | IPSL | CANESM5 | MPI-ESM | CANESM5 | CANESM5(3) |
| **ETR** | CANESM5 | MIROC6 | MIROC6 | INM | BCC | MIROC6 | MIROC(3) |
| **HWF** | All - BCC | ALL | ALL | ALL | ALL | ALL |  |
| **HWI** | MIROC6 | CANESM5 | CANESM5 | INM | MIROC6 | CANESM5 | CANESM5(3) |
| **HWML** | BCC | BCC | BCC/CANESM5/CNRM/INM/IPSL/MRI/UKESM | BCC | IPSL | BCC | BCC(5) |
| **HWTL** | BCC | MPI_LR | BCC/CNRM/INM/IPSL/MRI/UKESM | BCC | MRI | MPI_LR | BCC(3) |
| **HSF** | All -BCC | ALL | ALL | ALL | ALL | ALL |  |
| **HSML** | BCC | BCC | NORESM2 | CANESM5 | BCC | BCC | BCC(4) |
| **MCDD** | IPSL | CANESM5 | IPSL | IPSL | BCC | CANESM5 | IPSL(3) |
| **WDD** | IPSL | IPSL | IPSL | IPSL | BCC | IPSL | IPSL(5) |
| **WSDI** | BCC | IPSL | MRI | IPSL/MIROC6/MPI-ESM/MRI | BCC | CNRM/IPSL/MPI-ESM/MPI-LR/MRI | IPSL(3) |
| **Most suitable** | **IPSL(6), BCC(4)** | **CANESM5(4), BCC(3), IPSL(3)** | **IPSL(5), UKESM(3), INM(3)** | **BCC(3), IPSL(3), CANESM5(3)** | **BCC(8)** | **BCC(3), IPSL(3), CANESM5(4)** |  |
